# Therapeutic Potential of Herbal Medicines in Combating Particulate Matter (PM)-Induced Health Effects: Insights from Recent Studies

**DOI:** 10.3390/antiox14010023

**Published:** 2024-12-27

**Authors:** Aekkhaluck Intharuksa, Warunya Arunotayanun, Mingkwan Na Takuathung, Yaowatat Boongla, Siripat Chaichit, Suthiwat Khamnuan, Anchalee Prasansuklab

**Affiliations:** 1 Department of Pharmaceutical Sciences, Faculty of Pharmacy, Chiang Mai University, Chiang Mai 50200, Thailand; aekkhaluck.int@cmu.ac.th (A.I.); siripat.chaichit@cmu.ac.th (S.C.); 2 Kanchanabhishek Institute of Medical and Public Health Technology, Faculty of Public Health and Allied Health Science, Praboromarajchanok Institute, Nonthaburi 11150, Thailand; 3 Department of Pharmacology, Faculty of Medicine, Chiang Mai University, Chiang Mai 50200, Thailand; mingkwan.n@cmu.ac.th; 4 Clinical Research Center for Food and Herbal Product Trials and Development (CR-FAH), Faculty of Medicine, Chiang Mai University, Chiang Mai 50200, Thailand; 5 Department of Sustainable Development Technology, Faculty of Science and Technology, Thammasat University, Pathum Thani 12120, Thailand; yaowatat@tu.ac.th; 6 Faculty of Pharmacy, Western University, Pathum Thani 12150, Thailand; suthiwat.khamnuan@gmail.com; 7 College of Public Health Sciences, Chulalongkorn University, Bangkok 10330, Thailand; anchalee.pr@chula.ac.th; 8 Center of Excellence on Natural Products for Neuroprotection and Anti-Ageing, Chulalongkorn University, Bangkok 10330, Thailand

**Keywords:** air pollution, environmental hazards, herbal medicine, inflammation, oxidative stress, phytochemicals, particulate matter, PM, respiratory health

## Abstract

Particulate matter (PM), particularly fine (PM_2.5_) and ultrafine (PM_0.1_) particles, originates from both natural and anthropogenic sources, such as biomass burning and vehicle emissions. These particles contain harmful compounds that pose significant health risks. Upon inhalation, ingestion, or dermal contact, PM can penetrate biological systems, inducing oxidative stress, inflammation, and DNA damage, which contribute to a range of health complications. This review comprehensively examines the protective potential of natural products against PM-induced health issues across various physiological systems, including the respiratory, cardiovascular, skin, neurological, gastrointestinal, and ocular systems. It provides valuable insights into the health risks associated with PM exposure and highlights the therapeutic promise of herbal medicines by focusing on the natural products that have demonstrated protective properties in both in vitro and in vivo PM_2.5_-induced models. Numerous herbal medicines and phytochemicals have shown efficacy in mitigating PM-induced cellular damage through their ability to counteract oxidative stress, suppress pro-inflammatory responses, and enhance cellular defense mechanisms. These combined actions collectively protect tissues from PM-related damage and dysfunction. This review establishes a foundation for future research and the development of effective interventions to combat PM-related health issues. However, further studies, including in vivo and clinical trials, are essential to evaluate the safety, optimal dosages, and long-term effectiveness of herbal treatments for patients under chronic PM exposure.

## 1. Introduction

Airborne particulate matter (PM) has become a concern for humans due to its adverse health effects. The emission source, distribution, size, and chemical composition of particulate matter are important factors that can alter its impacts on health effects [1]. Recent research reports have indicated that fine and ultrafine particulate matter pose significant threats to the atmosphere. Both fine and ultrafine particles can originate from anthropogenic and natural sources, although these sources often differ. When it comes to the atmosphere, ultrafine particles, also referred to as PM_0.1_, consist of particles with an aerodynamic diameter of less than or equal to 0.1 μm [2,3]. These particles have become increasingly significant because they contribute to aerosols and environmental health problems that pose serious risks to public health [3,4]. Ultrafine particles can have detrimental effects on the nervous, circulation, cardiovascular, and respiratory systems [5,6]. Most studies have found that these particles are produced from forest fires [5,7] and biomass burning [8,9,10]. The phrases articulate matter 2.5 (PM_2.5_) and fine particles refer to particulate matter in the atmosphere with an aerodynamic diameter less than or equal to 2.5 μm [11]. PM_2.5_ is commonly generated from traffic [12,13,14] and industrial emissions [15]. Its chemical composition varies depending on sources of emission, photochemical oxidation processes, and meteorological conditions [15,16]. Although PM_0.1_ and PM_2.5_ particles are small in size, both are widely considered to be highly toxic. Fine and ultrafine particles typically enter the human body through inhalation, dermal contact, and ingestion pathways [17]. Outdoor PM inhalation has raised serious concerns, as the International Agency for Research on Cancer (IARC) has classified it as a human carcinogen. The inhalation of particulate matter (PM) is closely associated with oxidative stress, mitochondrial damage, lipid peroxidation, the upregulation of genes related to vascular inflammation, and early atherosclerosis [18]. These effects result from various cellular responses, such as the generation of reactive oxygen species (ROS, including O_2_ and OH), the induction of antioxidant defenses, pro-inflammatory responses, and, ultimately, cell death. These outcomes are triggered by the diverse physicochemical properties of particulate matter [18,19]. On the one hand, PM is a complex, heterogeneous mixture of inorganic ions, metals, organic matter, and polycyclic aromatic hydrocarbons (PAHs) and derivative PAHs. The physical structure, chemical composition, and source of these components differ significantly. Both toxicology and epidemiology continue to suggest that these components of PMs are probable causes of adverse effects on human health due to their ability to generate reactive oxygen species (ROS) in biological tissues [20]. While significant research exists, the mechanism underlying the long-term exposure of PMs, particularly ultrafine particles, remains poorly defined.

In this review, we gathered literature data on ultrafine particles, primarily identifying research articles related to their characteristics, size, morphology, emission sources, concentrations, distributions, exposure, and potential health impacts on humans. Additionally, we considered their toxicity, including the presence of PAHs, heavy metal compounds, and gaseous pollutants in the aerosol fraction. Secondary aerosol (SA) fractions are not regularly monitored in outdoor environments, especially in relation to ultrafine particles, despite being a significant contributor to environmental air pollution and toxicity. Therefore, it is essential to bridge the knowledge gap regarding secondary aerosols, their contributions to PM_2.5_ and PM_0.1_ concentration, their toxicity, and their impacts on human health. Many studies have demonstrated that the levels of secondary organic aerosols (SOA) [21,22,23] and secondary inorganic aerosols (SIAs) [24] have increased in association with air pollution. Secondary organic aerosols (SOAs) are formed through the atmospheric oxidation of both biogenic and anthropogenic gas-phase organic compounds (e.g., volatile organic compounds or VOCs). SOA, often quantified as the surrogate oxidized organic aerosol (OOA), typically constitutes a significant fraction of organic aerosols (OAs), particularly after prolonged atmospheric processing in urban areas. They are also found in downwind plumes. At urban and regional scales, emissions from on-road and off-road motor vehicles are prominent contributors to the observed concentrations of PM_2.5_ and organic aerosols (OAs) through both direct primary emissions and the release of reactive organic gasses that serve as precursors to secondary organic aerosols (SOAs) [22]. The main components of secondary inorganic aerosols (SIAs) include NH^4+^, SO_4_^2−^, and NO^3−^, which commonly exist as NH_4_NO_3_, NH_4_HSO_4_, and (NH_4_)_2_SO_4_. These SIAs are formed through complex chemical reactions involving SO_2_, NOx, and NH_3_ in the air, as well as droplets and particles. This review highlights the evidence, the need for recent research, its relevance, and its links to health impacts. In addition, a limited number of reports have investigated the vertical profiles of SOAs in the urban boundary layer (UBL) [25]. SOAs exhibit variations in their vertical distribution. This knowledge gap limits our understanding of SOA characteristics and their impacts.

Despite the efforts from various fields to address PM_2.5_-induced health challenges, no unified or standard approach has been established. The researchers have evaluated and reported the benefit of supplements and dietary interventions as protective strategies against PM_2.5_-induced health effects; however, a comprehensive review focusing on the potential of herbal medicines and their phytochemical properties in mitigating these adverse impacts is still lacking. This review fills this gap by collectively examining the therapeutic promise of natural products, emphasizing their antioxidant and anti-inflammatory mechanisms, and offering a detailed analysis of their potential to alleviate PM_2.5_-induced health damage across multiple physiological systems. These findings underscore the promising application of natural products as a foundation for future research and as a pathway through which to develop alternative therapeutic strategies to combat the pressing global challenge of PM-related health issues.

### 1.1. Emission Sources of Atmospheric Particulate Matter

**Figure 1:**
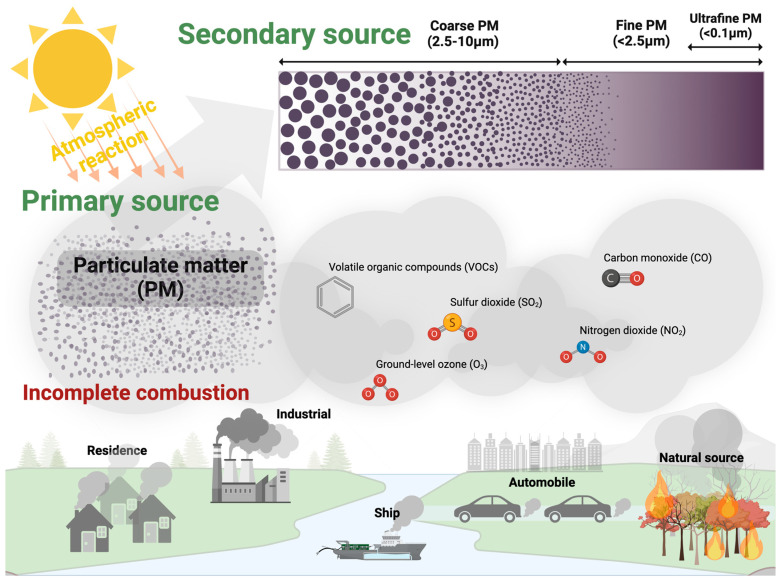
Sources of hazardous chemicals and particulate matter, the size distribution of PM, and the impact of PM on human health. Created using BioRender (Intharuksa, A., 2024). Available at https://BioRender.com/b97y489 (accessed on 20 November 2024).

For centuries, the sources and nature of ambient air pollution have been a cause for concern. Many studies have reported that the sources of primary particles include the combustion of coal [26,27], the combustion of wood and wood fuel [28,29], forest fires, eroded crustal material [30], windblown soil, and activities such as construction and demolition [31] (Figure 1). Other sources of particles include industrial dust [32], desert dust [33], unpaved roads, construction sites, soil erosion, sand, salts, sea surfaces, break and tire wear, windblown spray and foam, biological activities [34], volcanic eruptions, and meteoritic debris [35,36,37]. In urban areas, combustion is a major source of primary aerosol particles, including those formed from gasoline and diesel combustion [38], as well as oil emitted in vehicle exhaust [39,40,41]. Particulate matter (PM) is not a single air pollutant but rather a mixture of many chemical species. It is a complex mixture of solids and aerosols composed of small liquid droplets, dry solid fragments, and solid cores with liquid coatings. The properties of these particles generally correlate with their size, shape, and chemical composition. Some studies conducted in urban areas have reported on emission sources, distribution, characteristics, and behaviors, and also on chemical distinctions between particles smaller and larger than 1 μm. The aerodynamic diameter allows for the classification of PM into coarse (PM_10_), fine (PM_2.5_), and ultrafine (PM_0.1_) particles, which typically originate from different emission sources and possess distinct chemical compositions (Figure 1). PM_10_, or coarse particulate matter less than 10 μm in diameter, includes primary sources such as vehicle emissions, dust from construction sites, landfills and agriculture, wildfires, and brush/waste burning, industrial sources, wind-blown dust from open lands, pollen, and fragments of bacteria [34,42,43]. PM_2.5_ refers to particles with diameters smaller than 2.5 μm and is primarily composed of inorganic ions, carbonaceous compounds (such as black carbon (BC) and organic carbon (OC)), secondary organic aerosols (SOAs), volatile organic compounds (VOCs), polycyclic aromatic hydrocarbons (PAHs) and their derivatives, trace elements, and mineral dust. Primary emissions from events like forest fires and the burning of agricultural waste contribute to PM_2.5_, along with secondary sources resulting from chemical reactions involving precursor gasses in the atmosphere [44,45,46,47,48]. PM_0.1_, or ultrafine particulate matter with a diameter smaller than 0.1 μm [49], primarily originates from both natural and anthropogenic activities, including forest fires, biomass burning, diesel or gasoline combustion in vehicle engines, power plants, tobacco smoking, and ocean splashes [50,51,52,53,54]. Chemical analyses confirm that PM_0.1_ contains heavy metals, PAHs, and their alkylated (RPAHs), nitrated (NPAHs) and oxygenated (OPAHs) derivatives, all of which are known to be mutagenic and carcinogenic [55,56]. Additionally, PM_0.1_ contains BC, ionic compounds, elemental carbon (EC), and OC, which are toxic in nature [57,58]. Ultrafine particles are often formed through dynamic photochemical processes involving low-volatility gas compounds via nucleation, condensation, and coagulation [57,59]. Current research measures PM_10_, PM_2.5_, and PM_0.1_ using both stationary and mobile methods to assess environmental and exposure impacts [56,60]. During strong haze periods, commonly observed in the dry or winter season, research has reported significantly high levels of PM_10_, PM_2.5_, and PM_0.1_ [57].

### 1.2. Mechanism of Action of Particulate Matter on the Human Body at the Cellular and Molecular Levels

**Figure 2:**
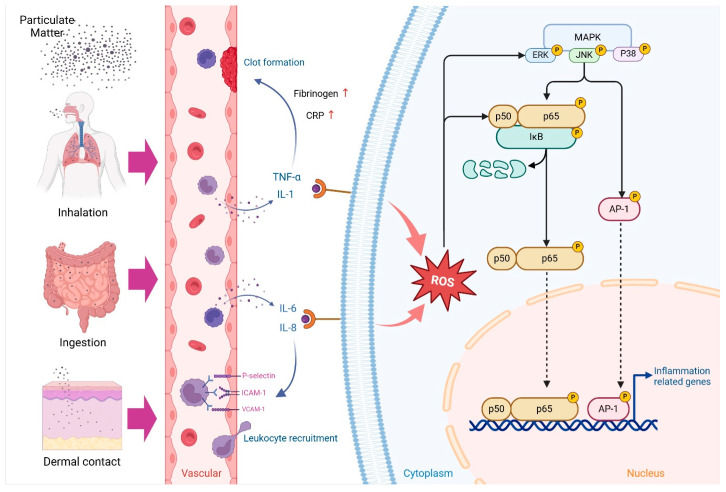
Key processes and molecular mechanisms underlying PM-mediated toxicity in human cells. Typically, PM can enter the human body through three major routes: inhalation, ingestion, and dermal absorption. Penetrated PM triggers inflammatory responses with the release of multiple proinflammatory cytokines as well as induction of intracellular ROS generation. Inside the cell, an elevated level of ROS further leads to activation of key signaling pathways related to inflamma-tion, particularly MAPKs and NF-kB, where solid arrow-headed black lines denote activation, dashed arrow-headed black lines indicate nuclear translocation, and solid right-angled blue ar-row represents gene transcription. Created using BioRender (Prasansuklab, A., 2025). Available at https://BioRender.com/b88q782 (accessed on 20 November 2024).

Particulate matter (PM) can enter the body through various pathways, including inhalation, ingestion, and dermal contact [17]. Inhaled PM often accumulates in the upper respiratory tract, specifically in the nasopharynx and oropharynx, while ingested PM adheres to the mucociliary clearance system, from where it may be swallowed and transported to the gastrointestinal tract [61]. Dermal exposure to PM can lead to skin penetration, especially through hair follicles or a disrupted stratum corneum [62,63]. Although PM penetration through the skin is possible, it typically remains on the surface, releasing soluble components that may contribute to harmful effects on the body [62,63,64]. The mechanisms underlying PM-induced toxicity involve several key processes (Figure 2): the generation of oxidative molecules and reactive electrophilic metabolites on the particle surface, the release of organic molecules and transition metals from the particles, the activation of an inflammatory cascade triggered by the particles, and a positive feedback loop in which ROS-activated inflammatory cells produce additional reactive oxygen and nitrogen species [65,66].

Oxidative stress triggered by PM_2.5_ is a fundamental molecular mechanism underlying its toxicity. Upon PM_2.5_ exposure, cells produce large amounts of reactive oxygen species (ROS), which influence cell signaling pathways (e.g., NF-κB, MAPKs, Keap1-Nrf2-ARE, and PI3K-Akt), affect ion channels and transport mechanisms (such as Ca^2+^ and mPTP), and modulate protein kinases, also impacting the ubiquitination and proteasome systems [67]. ROS affect the NF-κB pathway by inhibiting IκBα phosphorylation, modifying IKK (particularly via S-glutathionylation of IKKβ at cysteine 179), and interfering with IκB ubiquitination and degradation through Ubc12 inactivation. The mitogen-activated protein kinase (MAPK) signaling cascades, including extracellular signal-regulated kinase (ERK1/2), c-Jun N-terminal kinase (JNK), p38 kinase, and big MAP kinase 1 (BMK1/ERK5), function as central intracellular signal transduction pathways [68]. ERK activation, which is generally driven by growth factors (e.g., epidermal growth factor (EGF) and platelet-derived growth factor (PDGF)) and cytokine stimulation (e.g., IL-1β and TNF-α) via tyrosine kinase receptors [69], can also be initiated by ROS through the ligand-independent activation of EGF and PDGF receptors, thus activating Ras and the ERK pathway [70,71]. c-Jun N-terminal kinases (JNK), a critical subgroup within the mitogen-activated protein kinase (MAPK) superfamily, consist of three isoforms (JNK1, JNK2, and JNK3) encoded by separate genes [72]. The relationship between ROS and JNK activation is well established, yet the underlying molecular mechanisms of ROS-induced JNK activation are still being explored, involving pathways such as MAPKKK ASK1, Src kinase, and glutathione S-transferase Pi (GSTπ) [72]. Additionally, p38 is generally activated by inflammatory cytokines (e.g., tumor necrosis factor-alpha (TNF-α), IL-1β) and other stimuli, including hormones, G protein-coupled receptors, and environmental stresses (e.g., heat and osmotic shock) [73]. Certain proteins in the JNK pathway, such as ASK-1, also support p38 activation, with oxidative stress impacting proteins like ASK1, MEKK1-4, and MLK3, further activating p38 [67]. The Keap1-Nrf2-ARE pathway plays a vital role in maintaining cellular redox homeostasis and metabolism, initiating adaptive responses to oxidative stress that, if unregulated, may contribute to various inflammatory diseases [67]. The Keap1-Nrf2-ARE pathway is essential for maintaining cellular redox balance and metabolism, supporting adaptive responses to oxidative stress that, if unregulated, may contribute to inflammatory diseases. Under oxidative conditions, nuclear factor erythroid 2-related factor 2 (Nrf2) evades Kelch-like ECH-associated protein 1 (Keap1)-mediated degradation, translocates to the nucleus, and activates antioxidant response elements (ARE)-dependent genes encoding antioxidative and cytoprotective proteins, such as heme oxygenase-1 (HO-1), NAD(P)H dehydrogenase quinone 1 (NQO1), γ-glutamylcysteine synthetase, glutathione-related enzymes, and superoxide dismutase (SOD) [74]. Elevated intracellular ROS levels promote Nrf2 dissociation from Keap1 by oxidizing crucial cysteine residues (Cys273, Cys288, and Cys151) or by activating kinases (e.g., PKC, MAPK, phosphatidylinositide 3-kinases (PI3Ks), and protein kinase-like endoplasmic reticulum kinase (PERK)) that phosphorylate Nrf2 [75]. The phosphoinositide-3-kinase (PI3K)-Akt pathway, crucial for processes such as protein synthesis, cell cycle regulation, apoptosis, autophagy, and drug resistance, is generally activated by growth factors, hormones, and cytokines [76]. ROS directly activate phosphatase and tensin homolog (PTEN) while concurrently inactivating PTEN through cysteine oxidation, thereby enhancing Akt activation [67]. Additionally, ROS influence PTEN degradation through casein kinase II-mediated phosphorylation and transiently activate Akt by modulating its interaction with protein phosphatase 2A (PP2A) under low-ROS conditions [67]. The Ca^2+^ signaling network operates across a broad dynamic range to regulate cellular processes through the action of buffers, pumps, and exchangers located on the plasma membrane and within internal stores [77]. Intracellular Ca^2+^ modulates ROS production and clearance, shifting the redox state, while ROS influence Ca^2+^ signaling by oxidizing cysteine thiols on Ca^2+^ channels, pumps, and exchangers, such as RyR, IP3R, SERCA, PMCA, and NCX, which regulate channel gating and complex assembly [78]. ROS also impact the mitochondrial permeability transition pore (mPTP) and various kinases, including protein kinase A (PKA), protein kinase C (PKC), protein kinase D (PKD), receptor tyrosine kinases (RTKs), and Ca^2+^/calmodulin-independent protein kinase II (CaMKII) [67]. Additionally, ROS modulate the ubiquitination/proteasome system, which is integral to regulating the cell cycle, inflammatory and immune responses, protein folding, and endoplasmic reticulum-associated protein degradation [67].

PM exposure can trigger innate immune responses in monocytes and macrophages, leading to the production of reactive oxygen species (ROS) [79]. This, in turn, induces the release of pro-inflammatory cytokines, such as TNF-α and IL-6, by activated macrophages. These cytokines stimulate endothelial cells, increasing vascular permeability and facilitating the infiltration of immune cells into affected tissues. Cytokines such as interleukin-6 (IL-6) and interleukin-8 (IL-8) further promote leukocyte recruitment through interactions with endothelial cell adhesion molecules, including ICAM-1, VCAM-1, and P-selectin [80]. Pro-inflammatory pathways, such as nuclear factor-kappa B (NF-κB) and mitogen-activated protein kinases (MAPKs), the NOD-like receptor–related protein 3 (NLRP3) inflammasome, and activator protein 1 (AP-1), regulate the expression of inflammatory genes during this process [81]. These pathways are further amplified by cytokines. Additionally, leukocyte-derived reactive oxygen species (ROS) contribute to inflammation by inducing oxidative stress, which damages cells and releases additional pro-inflammatory signals. Arachidonic acid metabolites, including prostaglandins and leukotrienes, also play a crucial role in inflammation by promoting vasodilation and increasing vascular permeability. Systemically, TNF-α and IL-1 induce acute-phase responses, marked by the hepatic production of proteins such as C-reactive protein (CRP) and fibrinogen, which circulate in the plasma. Simultaneously, cytokines stimulate the coagulation cascade, leading to clot formation, fibrinolysis, and bradykinin production, all of which contribute to the regulation of vascular and immune responses. This interconnected cascade of cellular signals and mediators underscores PM’s role in inflammation and its potential to contribute to chronic inflammatory diseases.

## 2. Materials and Methods

This study was conducted by reviewing published information on particulate matter (PM)-related health issues and the potential of herbal medicines, sourced from books and peer-reviewed articles available up to December 2024. Key electronic databases utilized included Web of Science, Scopus, PubMed, Google Scholar, and Elsevier. The search terms were particulate matter, urban dust, PM_2.5_, PM_0.1_, herbal medicine, herbal extracts, herbs, plants, phytochemicals, and natural products. Data from in vitro, in vivo, and clinical studies of herbal medicine on PM-related health issues were collected. The extensive findings were subsequently analyzed and systematically summarized.

## 3. Results

### 3.1. Physiological Effects of Particulate Matter on Human Health

Air pollution, stemming from various sources, is currently a pressing environmental issue affecting diverse populations worldwide. While the health consequences of air pollution have gained global attention since the twentieth century [82], ongoing research continues to investigate the specific impacts of particulate matter (PM). Studies have demonstrated that PM exposure can occur through inhalation, skin contact, and ingestion, leading to a range of detrimental effects on the human body.

#### 3.1.1. Respiratory System Impact

The respiratory system serves as the primary entry point for PM into the body and is particularly susceptible to its harmful effects, as PM can be inhaled and penetrate deep into the lungs depending on its size. PM larger than 10 µm tends to deposit in the trachea, whereas PM_2.5_ can traverse the tracheobronchial tract, and PM_0.1_, existing as ultrafine particles, can penetrate deeper into the alveoli [83]. To eliminate these particles, both the innate and adaptive immune systems engage in various processes to manage and mitigate the effects of PM exposure [84]. The innate immune system directly stimulates macrophages and epithelial cells to produce TNF-α, Transforming Growth Factor β1 (TGF-β1), IL-6, and IL-8, which lead to the loss of cell adhesion proteins, such as adherens and tight junctions [85,86], resulting in cell barrier damage, impaired bronchial mucociliary function, and an increased risk of infections [87]. Moreover, the innate immune system generates ROS through mitochondrial respiration, NADPH oxidase, and xanthine oxidase system [88]. Additionally, adaptive immunity activates T cells, particularly type 2 T helper cells, causing the overproduction of pro-inflammatory cytokines, such as IL-5 and IL-13, which stimulate inflammation in the lung airways [89]. In the respiratory system, one of the main mechanisms involves PM generating ROS, especially PM_2.5_ and PM_0.1_, which contribute to inflammation via intracellular signaling pathways, such as MAPK, NF-κB, and activator protein 1 (AP-1). These pathways cause transcriptional changes that enable cells to respond to oxidative stress. The increase in ROS disrupts intracellular calcium homeostasis, resulting in elevated calcium concentrations, which subsequently lead to cell necrosis and apoptosis [83,86].

Pathological studies of exposure to PM_2.5_ and PM_0.1_ have implicated these particles in a variety of respiratory tract issues. Notably, PM_2.5_ can exacerbate respiratory diseases such as allergic rhinitis, asthma, and chronic obstructive pulmonary disease (COPD) [90] and has also been linked to the development of lung cancer [91]. Ambient PM_2.5_ has a detrimental effect on asthma exacerbation following short-term exposure, particularly in high-risk populations such as children [92]. The presence of asthma tends to increase the production of ROS, and the antioxidant defense system in the lungs is often insufficient to counteract this increase [88]. The pathology of COPD associated with particulate matter, particularly PM_2.5_, involves multiple mechanisms contributing to disease progression. PM_2.5_ generates ROS, leading to oxidative stress in lung tissues, which subsequently triggers airway inflammation mediated by crucial cytokines such as IL-5, IL13, and TNF-α. Additionally, PM_2.5_ exposure causes hypersecretion, alveolar damage, decreased lung function, emphysematous lesions, and airway inflammation, exacerbating preexisting conditions [93]. In the case of PM-induced lung cancer, exposure to PM_2.5_ is associated with an increased incidence and mortality of lung cancer due to the presence of various harmful substances, including heavy metals, volatile organic compounds (VOCs), and polycyclic aromatic hydrocarbons (PAHs), which are known carcinogens [94,95]. Long-term exposure to PM_2.5_ can result in chronic lung inflammation, DNA damage, the suppression of DNA repair, and the promotion of the replication of fragmented DNA [96]. These persistent responses can induce cellular changes and mutations, potentially increasing the risk of cancer. Overall, the respiratory system is highly susceptible to both acute and chronic health consequences associated with PM exposure, including an increased risk of respiratory diseases, the worsening of existing conditions, and long-term health deterioration.

#### 3.1.2. Cardiovascular System Impact

Exposure to fine PM ranging from 0.1 to 2.5 μm in diameter has been recognized as a significant environmental health risk, particularly due to its role in the development and exacerbation of cardiovascular diseases (CVDs) [97]. These fine particles, because of their small size, can penetrate deep into the respiratory system and enter systemic circulation, ultimately reaching various organs, including the heart and blood vessels [98]. The cardiovascular effects of PM are mediated through several pathophysiological mechanisms, including oxidative stress, inflammation, endothelial dysfunction, and autonomic nervous system imbalance [99,100]. Collectively, these processes contribute to the development and progression of cardiovascular conditions such as hypertension, atherosclerosis, myocardial infarction, and heart failure [101]. One of the primary mechanisms through which PM exerts its harmful effects on the cardiovascular system is oxidative stress. Fine PM generates ROS, leading to oxidative damage to cellular components, including lipids, proteins, and DNA [102]. This oxidative stress not only impairs normal cellular function but also triggers inflammatory responses that further contribute to cardiovascular pathology. The inflammation induced by PM exposure is characterized by the release of pro-inflammatory cytokines, such as TNF-α and interleukins, which promote endothelial dysfunction and the formation of atherosclerotic plaques [103]. Endothelial dysfunction is a critical event in the development of atherosclerosis, a leading cause of CVDs. PM exposure impairs the endothelium’s ability to produce nitric oxide (NO), a vasodilator that plays a key role in maintaining vascular health [104]. Reduced NO availability leads to increased vasoconstriction, elevated blood pressure, and the promotion of vascular remodeling, all of which contribute to the progression of atherosclerosis [104,105]. Furthermore, PM exposure has been linked to autonomic nervous system imbalance, exacerbating cardiovascular dysfunction by altering heart rate variability and increasing sympathetic nervous system activity [106]. In addition to these mechanisms, chronic exposure to PM_0.1–2.5_ can lead to vascular remodeling and the development of high-risk atherosclerotic plaques that are prone to rupture [107]. This increases the likelihood of acute cardiovascular events, such as myocardial infarction and stroke [101,108,109]. Studies have also demonstrated that PM exposure can induce hypertension and worsen pre-existing cardiovascular conditions, highlighting its role in the progression of cardiovascular disease [101]. At the molecular level, PM_0.1–2.5_ activates several signaling pathways that exacerbate cardiovascular damage. For instance, the activation of the NF-κB and JAK/STAT pathways by PM exposure leads to the sustained production of pro-inflammatory cytokines, which further aggravate endothelial dysfunction and promote the development of atherosclerosis [110,111]. Additionally, PM-induced oxidative stress can lead to mitochondrial dysfunction, further contributing to cellular damage and the progression of cardiovascular disease [100]. In conclusion, the physiological effects of PM_0.1–2.5_ on the cardiovascular system are profound and multifactorial. PM exposure triggers a cascade of harmful processes, including oxidative stress, inflammation, endothelial dysfunction, and autonomic imbalance, all of which contribute to the development and progression of cardiovascular diseases. These findings underscore the importance of reducing PM exposure as part of public health strategies aimed at mitigating the global burden of cardiovascular disease. Addressing air pollution and its cardiovascular impacts is crucial for improving health outcomes, particularly in populations exposed to high levels of fine PM.

#### 3.1.3. Skin Impact

PM is a significant air pollutant that poses considerable risks to skin health [112]. Due to their small size (PM_0.1–2.5_), these fine particles can penetrate deep into the skin, leading to various dermatological issues [113]. Their ability to carry harmful chemicals further exacerbates skin conditions. PM has been shown to penetrate the skin via transepidermal routes and absorption through hair follicles and sweat ducts [114]. The possible mechanisms by which PM affects the skin involve oxidative stress, inflammation, AhR activation, and alterations to the skin microbiome [115]. These processes may contribute to the development of various skin conditions, such as acne, hyperpigmentation, atopic dermatitis, and psoriasis [116,117]. One of the promising mechanisms by which PM affects skin health is oxidative stress. PM present in air pollution generates quinones, which are redox-cycling chemicals that produce ROS. The increased levels of ROS and free radicals within cells and their mitochondria overwhelm the skin’s antioxidant defenses, leading to the depletion of both enzymatic and non-enzymatic antioxidant capacities. Oxidative stress triggers a cascade of biological events, including genetic damage and the activation of transcription factors (AP-1 and NF-κB) and signaling pathways (ERK, JNK, and p38 MAPK). These processes contribute to cell growth, differentiation, and the degradation of dermal connective tissue [118,119]. PM exposure can induce the inflammatory cascade by promoting a pro-inflammatory environment in the skin, leading to elevated levels of IL-8 [120]. This cytokine plays a key role in activating granulocyte chemotaxis and enhancing phagocytosis. The inflammatory-related genes, including IL-1β and IL-6, are upregulated in skin tissues exposed to PM_2.5_ [119]. PM exposure elevates the expression of caspade-14, which affects the keratinocyte differentiation, and further impairs normal epidermal processes [121]. Ambient air pollution causes skin damage by activating the aryl hydrocarbon receptor (AhR), a transcription factor crucial for maintaining skin integrity and immune responses. AhR is expressed in various skin cells, including keratinocytes and melanocytes [122]. Pollutants in the air generate free radicals, triggering inflammatory processes in the skin that further activate AhR-dependent pathways and alter the skin microbiota [115]. Prolonged exposure to air pollution can activate AhR, contributing to extrinsic skin aging, wrinkle formation, and change in pigmentation [123]. The alteration of resident microflora on the skin is one factor in skin pathologies. PM has been shown to affect the skin microbiota, potentially disrupting its balance and contributing to skin disorders. Prolonged exposure to PAHs can change the microbial community on the skin [124] and influence the bacteria involved in the degradation of PAHs and other xenobiotics [125]. Pollutant exposure alters the composition of skin microorganisms, thereby affecting the prevalence and severity of atopic dermatitis [126]. In conclusion, the physiological effects of PM_0.1–2.5_ on skin health are intricate and multifaceted. PM exposure triggers a harmful cascade of processes, including oxidative stress, inflammation, AhR activation, and alterations in the skin microbiome. Collectively, these factors significantly contribute to the development of skin disorders. These findings underscore the importance of addressing air pollution as a risk factor for skin diseases.

#### 3.1.4. Neurological System Impact

PM, particularly fine particles classified as PM_0.1–2.5_, is increasingly recognized as a significant environmental factor contributing to neurological diseases [127,128]. These particles, due to their small size, can penetrate deep into the respiratory system, cross the blood–brain barrier (BBB), and exert a range of harmful effects on the central nervous system (CNS) [129]. The impact of PM on neurological health is multifaceted, involving mechanisms such as oxidative stress, inflammation, the disruption of the BBB, and direct neurotoxicity, all of which contribute to the development and progression of neurodegenerative diseases such as Alzheimer’s disease (AD), Parkinson’s disease (PD), and cognitive decline [100,128,130]. One of the primary mechanisms through which PM_0.1–2.5_ affects the nervous system is oxidative stress. Fine particulate matter generates ROS, which lead to cellular damage and contribute to neuroinflammation [131,132]. This oxidative stress is particularly harmful to neurons, which are highly susceptible to oxidative damage due to their high metabolic demand and limited antioxidant defenses [132,133]. Studies have shown that exposure to PM_2.5_ increases markers of oxidative stress and microglial activation in the brain, which are key processes in the pathogenesis of neurodegenerative diseases [134,135]. This oxidative stress not only damages neuronal cells but also exacerbates the inflammatory response, further contributing to neuronal injury and neurodegeneration [128,129]. Inflammation plays a critical role in the neurological effects of PM exposure. The inhalation of PM can trigger an immune response in the lungs, leading to the release of pro-inflammatory cytokines that enter systemic circulation and reach the brain [136]. Once in the CNS, these cytokines can activate microglia, the resident immune cells of the brain, leading to chronic neuroinflammation [128,137]. Chronic inflammation is a key factor in the progression of neurodegenerative diseases, contributing to neuronal death and the formation of pathological features such as amyloid plaques in AD and Lewy bodies in PD [129,138]. Moreover, studies have demonstrated that long-term exposure to air pollution is associated with increased levels of inflammatory markers in the brain and an accelerated decline in cognitive function [130]. Another significant mechanism through which PM_0.1–2.5_ impacts neurological health is by compromising the integrity of the BBB [139,140]. The BBB is a critical structure that protects the brain from harmful substances in the blood while allowing the passage of essential nutrients [141]. Prolonged exposure to PM can alter the expression of genes related to BBB function, increasing its permeability and allowing toxic substances to enter the brain. This disruption of the BBB can lead to an influx of harmful particles and inflammatory mediators into the CNS, exacerbating neuroinflammation and contributing to neurodegenerative processes [128,142]. In addition to these indirect effects, PM_0.1–2.5_ can also exert direct neurotoxic effects. Research indicates that fine particulate matter can induce apoptosis (programmed cell death) in neuronal cells and hinder the differentiation of neural stem cells, which are essential for neuron development and brain function [143]. Animal studies have further shown that exposure to PM_2.5_ can lead to brain damage, including neuron death and the formation of neurofibrillary tangles, a hallmark of AD [132,140]. Additionally, the inhalation of PM particles is associated with reduced neurogenesis in the hippocampus, a brain region critical for learning and memory [144]. The evidence linking PM exposure to neurological diseases is further supported by epidemiological studies [145,146]. For instance, research has shown that individuals living in areas with high levels of PM_2.5_ are at higher risk of developing cognitive decline, AD, and PD [130,145,147]. These findings are consistent across different populations, highlighting the global relevance of air pollution as a risk factor for neurological disorders [145,147]. Furthermore, the association between PM exposure and neurological diseases is not limited to neurodegeneration; it also extends to mental health disorders such as depression and anxiety [148]. Studies have found that exposure to air pollution is linked to increased rates of depression and anxiety, potentially due to the inflammatory and oxidative stress pathways triggered by PM [140,149]. In conclusion, the physiological effects of PM_0.1–2.5_ on the nervous system are profound and complex. PM exposure triggers a cascade of harmful processes, including oxidative stress, inflammation, the disruption of the BBB, and direct neurotoxicity, all of which contribute to the onset and progression of neurological diseases. These findings underscore the importance of addressing air pollution as a modifiable risk factor for neurological health. Public health strategies aimed at reducing PM exposure could play a crucial role in mitigating the burden of neurological diseases and improving the overall quality of life.

#### 3.1.5. Gastrointestinal System Impact

PM exposure, a significant environmental concern, is associated with adverse health effects, including the development of gastrointestinal (GI) disorders. The impact of PM varies by particle size: larger particles (>PM_10_) are restricted to the upper airway, while smaller particles (<PM_2.5_) can penetrate to the respiratory endothelium, circulate through the systemic system, and eventually accumulate in multiple organs [150,151]. PM may reach intestinal tissues through direct mechanisms (via digestion or the macrophage clearance of particles) or indirectly by inducing systemic inflammation [65]. PM exposure is implicated in the development of GI diseases such as inflammatory bowel disease (IBD) [152,153], appendicitis [154], gastroenteritis [155] and colorectal cancer [156,157]. While the precise mechanisms through which PM impacts the gastrointestinal system remain elusive, it is likely that oxidative stress plays a pivotal role. The effects of PM exposure may be attributed to disruptions in redox homeostasis, inflammation, and genotoxicity [158]. Exposure to PM_2.5_ generates ROS and induces lipid peroxidation, which can damage the gastrointestinal mucosa. This damage compromises tight junctions in the epithelial cells, facilitating bacterial invasion and triggering immune responses that may contribute to IBD, particularly ulcerative colitis [159,160,161]. Prolonged exposure to PM increases the expression of pro-inflammatory cytokines such as IL-6, IL-10, IL-18, IL-1β, and TNF-α, enhances small intestinal permeability, alters the composition and function gut microbiota, and ultimately induces inflammatory responses in the intestines [161,162]. The gut microbiota plays a crucial role in maintaining gut health. It is essential for various host functions, including immunity and metabolism, and it produces beneficial substances, particularly short-chain fatty acids [66]. Exposure to PM has been shown to significantly alter certain genera of gut microbiota, leading to dysbiosis [161,162]. These changes in the composition and function of gut microbiota may contribute to the development of several disorders, such as IBD and other inflammatory disorders of GI tract [163]. In conclusion, the physiological effects of particulate matter on the GI tract are complicated. Exposure to PM induces harmful processes, including oxidative stress, inflammation, the disruption of the epithelial barrier, and alterations in gut microbiota, all of which contribute to the development of GI diseases. These findings underscore the importance of addressing this environmental issue and highlight potential pathways for mitigating public impact, ultimately improving the clinical outcomes for affected populations.

#### 3.1.6. Ocular Impact

The eyes are constantly exposed to the external environment, with the corneal epithelium serving as a barrier to protect the ocular surface, making it susceptible to airborne pollutants. PM, especially PM_2.5_ and PM_10_ from air pollution, is associated with a range of corneal diseases, from mild irritation, dry eye syndrome (DES), and allergic conjunctivitis to more severe conditions such as infectious keratitis and retinal damage [164]. Several studies have revealed that chronic exposure to PM_2.5_ exerts toxic effects on the corneal and conjunctival epithelium. PM_2.5_ exposure induces cell damage in human corneal epithelial cells (HCECs) in a time- and dose-dependent manner by significantly reducing cell viability, migration, and proliferation, while also disrupting the cell cycle, increasing apoptosis, and altering mucin expression [164,165,166,167].

PM contains harmful components, including heavy metals, polycyclic aromatic hydrocarbons, and other organic compounds, that can penetrate the ocular surface. However, direct contact with PM_2.5_ is not the primary factor in PM_2.5_-induced corneal and conjunctival damage. Instead, critical mechanisms include the production of ROS, the release of pro-inflammatory cytokines such as NF-κB, TNF-α, granulocyte macrophage colony-stimulating factor (GM-CSF), IL-6, and IL-8, and the activation of autophagy [164,165,166,167]. The biological pathways activated by PM exposure contribute to a cascade of cellular stress and inflammatory responses in ocular tissues. Previous research has observed increased ROS production, which impairs the antioxidant defense mechanisms of ocular cells, resulting in cellular damage and apoptosis [168].

Animal- and cell-based studies simulate PM exposure to further understand its mechanisms and effects on ocular health. In rat models, prolonged exposure to PM_2.5_ disrupted the proliferation and differentiation of limbal stem/progenitor cells (LSPCs), which are essential for maintaining corneal epithelial homeostasis. This impairment led to corneal epithelial defects and a thinner epithelium in both the cornea and limbus. Similar effects were observed in humans residing in areas with high PM_2.5_ concentrations (above 35 μg/m^3^), who were found to have a thinner limbal epithelium, indicating a potential loss of LSPCs [169]. Tan et al. (2018) conducted experiments on mice, demonstrating that high levels of PM_2.5_ exposure significantly reduced tear production and disrupted tear film stability, leading to dry eye symptoms though NF-κB activation [165]. Similarly, Kang et al. (2020) observed that prolonged PM exposure in rats led to dry eye-like symptoms, including corneal damage, the thinning of the cornea, instability of the tear film, decreased tear production, retinal injury, and structural changes. PM exposure was notably associated with decreased thickness in the corneal epithelium and the nerve fiber layer (NFL)/ganglion cell layer (GCL), along with an increase in intraocular pressure—a known risk factor for glaucoma [170]. Additionally, Cui et al. (2018) found that exposure to PM_2.5_ significantly impaired corneal epithelial cell migration in both animal models of corneal abrasion and in cell cultures. This impairment was attributed to the suppression of FAK/paxillin phosphorylation and interaction in HCECs, reduced RhoA activity, and disrupted actin filament reorganization, leading to corneal epithelial defects and delayed wound healing [168].

Collectively, these findings underscore PM’s potential to impair ocular function and increase the risk of long-term eye diseases with chronic exposure. By activating multiple pathways, PM exposure initiates chronic inflammation that can contribute to eye conditions, including chronic dry eye and conjunctivitis, as well as degenerative diseases in the cornea. Notably, the disruption of cellular homeostasis triggers autophagy and apoptosis, suggesting that PM-induced stress not affects only the ocular surface but also extends to the deeper structures within the eye.

### 3.2. Herbal Medicines in Prevention and Treatment of Particulate Matter Exposure

As air pollution continues to be a significant global health concern, scientists are actively exploring various approaches to mitigate the adverse effects of PM exposure. Chronic exposure to PM leads to the release of cytokines like IL-6, IL-8, IL-1β, and TNF-α from inflammatory cells, further exacerbating inflammation. This inflammatory response stimulates the production of ROS, resulting in lipid peroxidation and further tissue damage [171,172]. As a result, PM exposure affects multiple organ systems, contributing to various health complications. In addition to technological solutions, such as air purification systems, there is growing interest in the potential of natural products to provide protective effects against PM-related health risks. PM contains harmful chemicals that can trigger oxidative stress and inflammatory responses, making the exploration of herbal medicines a promising area of research.

Herbal medicine and phytochemicals with anti-inflammatory and antioxidant properties have drawn attention for their potential to prevent and alleviate PM-induced health impacts. For example, *Curcuma longa* L. (turmeric) is well-known for its potent antioxidant, anti-inflammatory, antimicrobial, and anticancer properties, making it a promising candidate for PM-related health protection. The ability of turmeric to scavenge free radicals and inhibit key inflammatory mediators such as cyclooxygenase-2 (COX-2), lectin-like oxidized low-density lipoprotein receptor-1 (LOX-1), inducible nitric oxide synthase (iNOS), and pro-inflammatory cytokines such as IFN-γ, TNF, IL-1β, and IL-6, along with its ability to suppress transcription factors NF-κB and AP-1, highlighted its significant role in reducing inflammation associated with PM exposure [172,173,174,175]. An in vivo study demonstrated that administering a *Curcuma longa* extract at a dosage of 3 mg/kg daily for 30 days, following exposure to soot particles at a concentration of 1064 mg/m^3^ for 8 h, significantly reduced levels of TNF-α and IL-6. Additionally, the treatment decreased malondialdehyde (MDA) levels, an oxidative stress marker produced during lipid peroxidation, which is triggered by ROS from soot exposure [172]. At the DNA level, Dendrobium officinale water extract (DOWE) demonstrates a protective role against PM_2.5_ -induced DNA damage and inflammation in human peripheral blood lymphocytes (hPBLs). In the comet assay, DOWE showed concentration-dependent effects in reducing the olive tail moment, a marker of DNA fragmentation. This effect is likely attributed to the strong radical-scavenging properties of *D. officinale* polysaccharides [176]. Additionally, DOWE significantly suppressed the release of pro-inflammatory cytokines, including IL-1β, IL-2, and TNF-α, which were elevated due to PM_2.5_ exposure [177].

In addition to turmeric and DOWE, other plant extracts and phytochemicals are being investigated for their protective effects against PM-related health risks across various systems, offering a more comprehensive understanding of their mechanisms and potential applications, which are categorized by system in the sections below.

#### 3.2.1. Herbal Medicines for Respiratory Systems

Decades of epidemiological research have consistently demonstrated the existence of a positive correlation between prolonged exposure to PM and an elevated risk of developing chronic respiratory diseases, including asthma, pulmonary dysfunction, pneumonia, and lung cancer [178]. Considering the ongoing environmental challenges associated with PM, discovering innovative preventive or therapeutic interventions to shield the human respiratory system is vital for diminishing the morbidity of chronic respiratory diseases. Phytochemicals, plant extracts, and traditional herbal formulation have shown promise in this regard. The potential of various phytochemicals to prevent and manage the pathologies associated with PM exposure has been extensively explored in both in vitro and in vivo studies, as detailed in Table 1. Curcumin, the bioactive compound isolated from *Curcuma longa* L., is renowned for its antioxidant, anti-inflammatory, and cardioprotective activities [179]. Studies have explored the efficacy of curcumin in mitigating the detrimental effects of PM_2.5_ and PM_10_ exposure in in vitro and in animal models [180,181,182,183]. In vitro studies have revealed that curcumin protects microvascular endothelial and bronchial epithelial cells by inhibiting apoptosis, reducing oxidative stress, and suppressing the release of pro-inflammatory cytokines, including TNF-α, IL-5, IL-8, and IL-12 [182,183]. The in vivo findings align with the in vitro data, suggesting that curcumin’s anti-inflammatory and antioxidant effects are mediated by the inhibition of NF-κB activation via the suppression of MAPK signaling pathways and the activation of Nrf2 [180]. Huang et al. demonstrated that curcumin could alleviate PM_2.5_-induced lung injury [181] by reducing oxidative stress through a decrease in MDA levels and enhancing the antioxidant defense system by increasing GSH-PX, T-AOC, and CAT activities. Additionally, curcumin suppresses the production of pro-inflammatory cytokines, including IL-1 and TNF-α. Based on the promising properties of curcumin, researchers have developed hollow mesoporous silica nanoparticle (HMSN)-based ROS-responsive nanoscale drug delivery systems to synergistically induce ROS clearance and anti-inflammation, thereby improving the treatment of acute lung injury [184].

Besides individual phytochemicals, herbal extracts have also been investigated for their potential to prevent and treat PM-induced health conditions, as shown in Table 2. Korean researchers have studied the efficacy of traditional medicinal herbs like *Citrus × junos* Siebold ex Yu. Tanaka [185,186], and Siegesbeckiae Herba [187,188] in addressing PM-related health issues. *Citrus × junos*, commonly known as yuzu, is highly regarded by consumers for its appealing flavor and rich content of phytochemicals, particularly flavonoids such as naringin, hesperidin, narirutin, and neohesperidin [189]. These bioactive compounds have established yuzu as a valuable source of dietary antioxidants, with numerous studies exploring its potential in the prevention and treatment of various diseases. Lee and colleagues conducted a study on the protective and alleviating effects of yuzu peel against health impacts from PM exposure, with promising results as outlined in Table 2 [185]. Similarly, Huang et al. investigated the effects of a water extract derived from yuzu waste byproducts (including peel, pulp, and seed) by examining its ameliorative properties (administered at 100 and 200 mg/kg/day for 7 days) on PM_10_-induced lung damage (10 mg/kg, intranasally for 6 h) in BALB/c mice [186]. The study found that the yuzu extract significantly attenuated PM_10_-induced pulmonary damage and inflammatory cell infiltration in the mouse model. It also markedly reduced key protein markers involved in inflammatory signaling pathways, including AKT, ERK, JNK, and NF-κB phosphorylation induced by PM_10_. Additionally, the extract reduced the production of ROS and nitric oxide in a dose-dependent manner in cellular models. It selectively enhanced enzymes involved in GSH synthesis, regeneration, and conjugation, increased cellular GSH levels, and mitigated the oxidation caused by PM_10_ exposure. Studies have explored the synergistic effects of plant extracts and probiotics in addressing the health consequences of PM exposure. In an in vitro study, a 70% ethanol extract of germinated *Rhynchosia nulubilis* fermented with *Lactobacillus pentosus* SC65 demonstrated protective effects against PM-induced cell death in type II alveolar epithelial A549 cells by reducing the levels of ROS, active caspase-9, caspase-3, and PARP proteins, while simultaneously increasing the expression of the antiapoptotic protein BCL-2 [190]. Jung et al. investigated the effects of a combination of *Lactobacillus casei* HY2782 and *Pueraria lobata* root extract in mitigating PM_2.5_-induced airway inflammation [191]. The study found that this combination significantly reduced the presence of Th2/Th17-derived cytokines (IL-4, IL-5, IL-13, IL-17A), immunoglobulin E, and leukotriene C4 in bronchoalveolar lavage fluid and serum, key contributors to allergies and asthma. In 2024, a water extract of *Chenopodium formosanum* Koidz, fermented with *Rhizopus microsporus* var. *oligosporus* BCRC 31996, that is rich in gluconic acid, uridine, pantothenic acid, L-pyroglutamic acid, L-(−)-malic acid, and acetyl-L-carnitine was studied for its effects on PM_2.5_-induced inflammation in alveolar macrophages [192]. The extract was found to restore cell viability to 76%, reduce ROS production, and decrease p-NFκB activation by up to 38%. Alongside plant-derived herbs, fungal and algal extracts have also been investigated for their potential applications. A protein extracted from *Ganoderma microsporum* was found to reduce the risk of neurological disorders in both mother rats and their offspring [193]. This was achieved by lowering white blood cell counts, alleviating inflammatory responses, mitigating PM_2.5_-induced memory impairment, and preventing dendritic branch decline in the hippocampus, and also by modulating microRNAs induced by PM_2.5_ exposure. Another species from the same genus, the water fraction of *Ganoderma formosanum* mycelium extract, was studied for its effects on PM-exposed murine alveolar macrophages by measuring ROS levels [194]. The extract demonstrated a reduction in intracellular ROS and an increase in cell viability in response to PM particles. Additionally, the *G. formosanum* extract reduced the p-NF-κB/NF-κB protein ratio and decreased the expression of inflammatory genes, such as IL-1β, IL-6, and TNF-α. An edible brown algae, *Sargassum horneri* (Turner) C. Agardh, known for its bioactive components such as polyphenols, alginic acid, polysaccharides, and chromene, has been shown to reduce the PM-exacerbated mRNA expression of pro-inflammatory cytokines (IL-1β, TNF-α, TGF-β, IL-6), pro-allergic cytokines (TSLP, IL-25, IL-33), and the chemokine IL-8 in the lungs of asthmatic mice [195]. Due to its anti-inflammatory properties, the 70% ethanol extract of *S. horneri* was investigated to assess the anti-inflammatory mechanisms in PM-induced MH-S lung macrophages [196]. This study revealed that the extract suppressed the secretion of inflammatory cytokines and decreased the expression of proteins such as PGE2, TNF-α, IL-6, IL-1β, NF-κB, and MAPKs in PM-activated macrophages. Additionally, it inhibited the elevated mRNA expression levels of TLR2, TLR3, TLR4, and TLR7 in PM-induced MH-S cells. Another study evaluated the anti-inflammatory properties of the *S. horneri* water extract in an in vivo mouse asthma model following PM exposure [197]. The findings showed reduced phosphorylation levels of MAPKs, the decreased expression of iNOS and COX2, and the lower mRNA expression of pro-inflammatory cytokines.

Apart from individual herbs, studies suggest that synergistic combinations of natural products may offer clinical benefits in terms of alleviating respiratory symptoms. Such mixtures have been used to treat various diseases for centuries, with their physiological advantages supported by traditional usage and empirical evidence [198]. Using acetone extracts, curry powder, which contains ingredients such as clove, turmeric, ginger, black pepper, chenpi, cumin, coriander, fenugreek, cardamom, nutmeg, fennel, and cinnamon, was investigated for its effects on pro-inflammatory responses and the production of both extracellular and intracellular ROS induced by the PM_2.5_ component, diesel exhaust particles (DEPs) [199]. The results showed that the curry powder extract inhibited DEP-induced IL-6 release and reduced extracellular ROS levels. In 2020, Saba and colleagues evaluated the effects of a combination of *Panax ginseng* and *Salvia plebeia* R. Br extract, prepared using 30% alcohol/water, in a mouse model of airway inflammation induced by coal fly ash [200]. The study found that, in in vitro experiments, the extract inhibited nitric oxide production and reduced the expression of pro-inflammatory mediators and cytokines (iNOS, COX-2, IL-1β, IL-6, and TNF-α) through the NF-κB and MAPK pathways in CFA-induced inflammation in MH-S cells. In another study, an extract of a mixture of *Lonicera japonica*, Arctic Fructus, and Scutellariae Radix, prepared with 30% alcohol, was studied for its effects in terms of relieving acute bronchial and pulmonary inflammation in an animal model exposed to PM_2.5_ [201]. The findings showed that the extract improved acute bronchial and pulmonary inflammation by inhibiting inflammatory cytokines through the NLRP3/caspase-1 pathway. Moreover, an ethanol extract combining *Ecklonia cava* and *Chrysanthemum indicum* was studied for its effects in terms of alleviating PM_2.5_-induced lung inflammation [202]. The extract was found to suppress the phosphorylation of MAPKs, including JNK, ERK, and p38, which regulate MUC5AC gene expression. It exhibited protective effects against PM_2.5_-induced pulmonary damage by reducing inflammatory cell infiltration and mucus secretion in lung tissues. Additionally, the extract decreased the levels of key inflammatory cytokines, TNF-α and IL-6, both in the serum and lung tissue of the PM_2.5_-induced pulmonary inflammation mouse model.

Recently, the potential of traditional herbal remedies from various medical systems, including Traditional Chinese Medicine, Traditional Korean Medicine, and Ayurvedic Medicine, to prevent and treat respiratory symptoms induced by PM exposure has been explored. These remedies are renowned for their effectiveness in addressing respiratory conditions and reducing inflammation. Examples include Bufei Huoxue, Deng-Shi-Qing-Mai-Tang, Gamgil-tang, Gu-Ben-Zhi-Ke-Zhong-Yao, Guo-Min, Yu-Ping-Feng, Ma Xing Shi Gan, Shengma, YiQiFuMai, and Zangsiwei in Traditional Chinese Medicine; Banhahubak, Biyeom-Go, Gwaruhaengryeon-hwan, and Kyung-Ok-Ko in Traditional Korean Medicine; and Chyawanprash in Ayurvedic Medicine (Table 3). Scientists have been working to design safe and effective herbal formulas to combat PM-induced lung injury. Zhang et al. found that a formula composed of *Citri Exocarpium Rubrum*, *Lablab Semen Album*, *Atractylodis Macrocephalae Rhizoma*, *Mori Folium*, and *Polygonati Odorati Rhizoma* effectively prevented PM-induced lung damage in mice [178]. This was achieved by increasing the activity of SOD and GSH-Px, reducing MDA and ROS levels, and attenuating the upregulation of pro-inflammatory cytokines (IL-6, IL-8, IL-1β, and TNF-α). The formula also downregulated the protein expression of NF-κB, STAT3, and Caspase-3. In addition to in vitro and animal studies, clinical research has also been conducted. In 2022, Chen and colleagues investigated the role of Qianjinweijing decoction, a Chinese herbal formulation, in alleviating PM_2.5_-induced lung dysfunction through a randomized, double-blind, placebo-controlled trial involving adults exposed to PM_2.5_ [203]. The results showed that the decoction alleviated PM_2.5_-induced lung dysfunction by reducing the percentage of restrictive ventilatory defects and the proportion of eosinophils in induced sputum, suggesting its potential as a treatment for air pollution-related chronic respiratory diseases. Another Chinese herbal formulation made of Qiju granules, based on the traditional Qi-Ju-Di-Huang-Wan formula (Lycium, Chrysanthemum, and Rehmannia), was studied in a randomized, double-blind, placebo-controlled trial involving 47 healthy college students exposed to PM_2.5_ [204]. The study found that Qiju granules may be effective in protecting lung function from the harmful effects of PM_2.5_. In Ayurvedic medicine, Hingu-Pippali Yoga was investigated in a randomized clinical study focusing on respiratory disorders among traffic police officers caused by air pollution [205]. The study revealed a significant reduction in symptoms such as coughing, rhinitis, and dyspnoea, along with notable improvements in pulmonary function tests, indicating increased lung capacity. A significant decrease in eosinophil count and ESR was also observed. These findings suggest that Hingu-Pippali Yoga is effective at reducing air pollution-induced respiratory disorders and enhancing lung capacity.

**Table 1:** Phytochemicals targeting PM-induced respiratory symptoms in in vitro and in vivo studies.

Phytochemicals	Dose and Administration	Model	PM Types and Administration	Biological Results
Aesculetin [206]	10 mg/kg, oral gavage	male BALB/c mice	PM_10_, inhale, 6.0 μg/mL	- attenuating airway thickening and mucus hypersecretion by inhibiting pulmonary inflammation via oxidative stress-stimulated TLR4 and EGFR
Astragaloside IV [207]	50–100 mg/kg, intraperitoneal injection	male Sprague Dawley rats	PM_2.5_, intratracheal instillation, 7.5 mg/kg	- mitigating lung injury by regulating the activity of TLR4/MyD88/NF-κB signaling pathway, - reducing inflammatory and oxidative stress responses.
Berberine hydrochloride [208]	50 mg/kg, oral gavage	male C57BL/6 mice	PM_2.5_, tracheal drip, 8.0 mg/kg	- activating the PPARγ signaling pathway - increasing ROS expression, upregulating protein levels of IL-6, IL-1β, TNF-α - regulating gene expression of STAT3 and SOCS3 - promoting collagen deposition - upregulating gene expression of fibrosis markers (TGF-β1, FN, α-SMA, COL-1, and COL-3) - downregulating E-cadherin expression
Betulinic acid derivative [209]	2–8 mg/kg, intra-gastric route	male mice	PM_2.5_, intranasal, 25 μL	- suppressing the induction of AKP and ALB levels in mouse lung tissues - reversing the suppression of SOD activity and the increase in NOS levels - enhancing levels of TNF-α and IL-6 - blocking acute inflammatory exudate, damage to alveolar septa, and infiltration of inflammatory cells
Chebulic acid [210]	5–10 μM	pulmonary alveolar epithelial NCI-H441 cell	urban particulate matter, 10–100 μg/mL	- reducing ROS - enhancing barrier integrity by safeguarding junctional parameters in pulmonary alveolar epithelial cells
Cudratricusxanthone O [211]	29–290 μg/kg, peroral	male Balb/c mice	PM_2.5_, 1 mg/kg, intratracheal instillation	- neutralizing ROS and blocked the ROS-induced activation of p38 MAPK - activating Akt, which helps to maintain endothelial integrity - decreasing vascular protein leakage, leukocyte infiltration, and release of pro-inflammatory cytokines into the bronchoalveolar lavage fluid
Curcumin [180,181,182,183]	50 mg/kg, peroral [180]	male C57BL/6N mice	PM_10_, 10 mg/kg, intratracheal instillation	- reducing alveolar damage - diminishing immune cell infiltration, and lowering pro-inflammatory cytokine production in both lung tissue and BALF - blocking the production of pro-inflammatory cytokines by inhibiting NF-κB activation through the suppression of MAPK signaling pathways - mitigating oxidative stress by activating Nrf2 and its downstream antioxidant signaling pathways
	100 mg/kg, intraperitoneal injection [181]	male Kunming mice	PM_2.5_, 20 mg/kg, intratracheal instillation	- elevating the expression of the HO-1/CO/P38 MAPK protein in lung tissue - decreasing the levels of ALB, LDH, and ALP in BALF - lowering the levels of MDA, IL-1, and TNF-α in lung tissue - enhancing GSH-PX, T-AOC, and CAT levels
	0–50 μM [182]	human microvascular endothelial (HMEC-1) cells	PM_2.5_, 0–600 μg/mL	- decreasing cell apoptosis and intracellular caspase 3 activity in HMEC-1 cells - decreasing levels of oxLDL, TNF-α, and IL-8 - stimulating the expression of NF-κB, cell adhesion molecule 1, and vascular cell adhesion protein 1.
	0–50 μM [183]	human bronchial epithelial BEAS-2B cells	PM_2.5_, 0–100 mg/mL	- decreasing apoptosis in BEAS-2B human bronchial epithelial cells by lowering the levels of intercellular ROS - elevating the expression of NRF2 - stimulating the expression of IL-5 and IL-13
Ginsenoside Rg1 [212]	100, 200 or 400 μg/mL	A549 human alveolar type II epithelial cells	PM_2.5_, 200–1200 μg/mL	- diminishing the A549 cell viability - decreasing LDH leakage and MDA generation in a concentration-dependent manner
Hyperoside [213]	20 mg/kg, intraperitoneal injection	human bronchial epithelial cells (Beas-2b cells) and male Balb/c mice	PM_2.5_, 10 mg/kg, nasal drop	- downregulating the autophagy markers, apoptotic proteins, and p-AMPK expression, and upregulating p-mTOR expression - alleviating pathological lung injury, lowered levels of PM-induced inflammatory cytokines (TNF-α and IL-6) - reducing the number of total cells in the BALF by inhibiting AMPK/mTOR signaling
Jujuboside B [214]	0.1–0.8 mg/kg, intravenous injections	BALB/c mice	PM_2.5_, 1 mg/kg, intratracheal instillation	- decreasing histological lung damage and the lung wet/dry weight ratio - decreasing autophagy dysfunction, apoptosis, inflammatory cytokine levels, and the number of lymphocytes in the bronchoalveolar fluid
Mogroside V [215]	100 nM	porcine alveolar macrophage 3D4/21 cells	PM_2.5_, 1 mg/mL	- blocking nitric oxide (NO) production and restored arginase activity - reducing phosphorylation of NF-κB p65 and expression of NLRP3 - decreasing intracellular ROS
Mojabanchromanol [216]	3.9–250 μg/mL	mouse alveolar type II epithelial MLE-12 cells	PM_2.5_, 31.3–62.5 μg/mL	- reducing levels of malondialdehyde and 8-hydroxydeoxyguanosine - suppressed PM-triggered TLR2/4/7 activation - decreasing ROS-mediated phosphorylation of MAPK, Erk1/2, and JNK - blocking the secretion of pro-inflammatory cytokines (IL-6, IL-1β, and IL-33)
Nootkatone [217]	90 mg/kg, oral gavage	BALB/c mice	diesel exhaust particles, 30 μg, intratracheal injection	- preventing the DEP-induced rise in airway resistance, lung inflammation, oxidative stress, and subsequent DNA damage and apoptosis by inhibiting NF-κB activation
Resveratrol [218]	50–100 mg/kg, oral gavage	male C57BL/6J mice	PM_2.5_, inhalation exposure for 5 months	- eliminating lung inflammation and fibrosis - inhibiting the autophagic process and NLRP3 inflammasome activation - inhibiting the autophagic process and NLRP3 inflammasome activity - decreasing IL-1β production in BEAS-2B cells
Rosavin [219]	50–100 mg/kg, intraperitoneal injection	male Sprague Dawley rat	PM_2.5_, 7.5 mg/kg, intratracheal instillation	- decreasing levels of tissue iron, malondialdehyde, and 4-hydroxynonenal, while increasing glutathione levels - increasing the expression of Nrf2 and other proteins related to ferroptosis
Ruscogenin [220]	0.1–1 mg/kg, oral gavage	male ICR mice	urban particulate matter, 50 mg/kg, intratracheal instillation	- reducing pathological injury, lung edema, vascular leakage, and VE-cadherin expression in lung tissue - lowering IL-6 and IL-1β levels, as well as NO and MPO levels, in both BALF and serum - suppressing the phosphorylation of NF-κB p65 and the expression of TLR4 and MyD88 in a dose-dependent manner
Salidroside [221]	50–100 mg/kg, intraperitoneal injection	male C57BL/6 mice	PM_2.5_, 7.5 mg/kg, intratracheal instillation	- decreasing mortality within 120 h and alleviated inflammatory responses by lowering the release of TNF-α, IL-1β, and IL-18 - blocking apoptosis and pyroptosis that led to tissue damage by regulating the Bax/Bcl-2/caspase-3 and NF-κB/NLRP3/caspase-1 signaling pathways.
Salvianolic acid B [222]	0.3–1.8 mg/kg, spraying into the exposure tower with a jet nebulizer	male ICR mice	PM_2.5_, 10 μg, intratracheal instillation	- protecting against airway inflammation and oxidative stress by inhibiting the TLR4/MyD88/TRAF-6/NLRP3 pathway and its downstream signals, ERK1/2 and P38
Sipeimine [223,224]	15–30 mg/kg, intraperitoneal injection [223]	male Sprague Dawley rat	PM_2.5_, 7.5 mg/kg, intratracheal instillation	- alleviating lung injury and decreasing pulmonary edema, inflammation and TNF-α and IL-1β levels in the BALF - enhancing the GSH expression - decreasing the 4-HNE, tissue iron, and MDA levels - restoring the downregulation of proteins associated with ferroptosis, including Nrf2, GPX4, HO-1, and SLC7A11
	15–30 mg/kg, intraperitoneal injection [224]	male Sprague Dawley rat	PM_2.5_, 7.5 mg/kg, intratracheal instillation	- enhancing the repair of lung tissue damage, reduced the inflammatory response, and suppressed pyroptosis in lung tissue - preventing the increase in pyroptosis-related proteins levels, including IL-1β, cleaved IL-1β, and GSDMD-N, and membrane pore formation and mitochondrial swelling - blocking the activation of the NLRP3 inflammasome, as indicated by the increased levels of NLRP3, cleaved caspase-1, and ASC proteins. - impeding by the NLRP3 activator nigericin
Sparstolonin B [225]	0.04–0.4 mg/kg, intravenous injection	Balb/c mice	PM_2.5_, 10 mg/kg, intranasal injection	- decreasing pathological lung injury, the lung wet/dry weight ratio, and levels of permeability - reducing myeloperoxidase activity in lung tissue - lowering inflammatory cytokine levels - suppressing the presence of lymphocytes in bronchoalveolar fluid - suppressing the expression of PM_2.5_-stimulated TLR2, TLR4, MyD88, and autophagy-related proteins, LC3II and Beclin 1
Stemonine [226]	45–180 mg/kg, oral	male Kunming mice	PM_2.5_, 40 mg/kg, intranasal instillation	- mitigating lung injury by reducing the levels of enzymes and cytokines related to inflammation and oxidative stress in a dose-dependent manner - enhancing the levels of SOD in a dose-dependent manner
Tubeimoside I [227]	45–180 mg/kg, oral	male BALB/c mice	PM_2.5_, 40 mg/kg, nasal instillation	- reducing the progression of PM_2.5_-induced pulmonary injury - reducing cytotoxic effects, levels of inflammatory mediators (TNF-α and IL-6) and oxidative damage
Ursolic acid [228]	20 mg/kg, oral gavage	female Sprague Dawley rat	PM_2.5_, 200 mg/m^3^, inhalation exposure for 30days	- decreasing goblet cell hyperplasia and collagen deposition in the nasal mucosa - lowering the expression of MUC5AC

**Table 2:** Plant extracts targeting PM-induced respiratory symptoms conducted in vitro and in vivo studies.

Sources	Part Uses	Solvent	Dose and Administration	Model	PM Types and Administration	Biological Results
*Adenophora stricta* Miq. (Campanulaceae) [229]	RT	water	100–400 mg/kg, oral	Balb/cAnNCrlOri mice	PM_2.5_, 1 mg/kg, intranasal instillation	- decreasing congested region of the lung tissue - preventing apoptosis and matrix degradation, and alleviating mucus stasis - blocking the influx of immune cells into the lungs - lowering pro-inflammatory cytokines and chemokines expression in the lungs - inhibiting the generation of ROS and lipid peroxidation
*Alpinia officinarum* Hance (Zingiberaceae) [230]	RZ	75% ethanol	50–200 mg/kg, oral	Kunming mice	PM_2.5_, 40 mg/kg, intranasal instillation	- decreasing the concentrations of albumin, lactate dehydrogenase, and alkaline phosphatase in BALF - reducing the levels of oxidative stress markers, NO, MDA, and NOS activity - lowering expression of IL-6 and TNF-α
*Asarum sieboldii* Miq. (Aristolochiaceae) [231]	RT	essential oil	0.0002–0.02%, nasal inhalation	female BALB/c mice	PM_10_, 100 μg in 50 μL of normal saline	- blocking the accumulation of goblet cells in the airways, leading to reduced epithelial thickness - decreasing collagen deposition in the lung tissues - blocking the influx of inflammatory cells into the BALF and decreasing the expression of serum IgE, IgG2a, and cytokines in the lung tissues
*Bletilla striata* (Thunb.) Rchb.f. (Orchidaceae) [232]	TB	95% ethanol	1.25–40 μg/mL	mouse macrophage cell line RAW264.7	PM_2.5_, 12.5–200 μg/mL	- lowering expression of inflammatory cytokines in macrophages - reducing pro-inflammatory protein expression - decreasing the NF-κBp65, IκB-α, JNK, ERK, and p38 levels
*Citrus × junos* Siebold ex Yu. Tanaka (Rutaceae) [185]	PL	distilled water	100–400 mg/kg, oral	male BALB/c mice	PM_10_, 100 mg/kg, intranasal instillation	- downregulating the levels of pro-inflammatory cytokines and NF-κB/apoptosis signaling-related markers
*Dioscorea batatas* Decne. (Dioscoreaceae) [233]	PL	95% ethanol	27–135 μg/kg, oral	male Balb/c mice	PM_2.5_, 1 mg/kg, intratracheal injection	- phenanthrene showed a marked ability to scavenge PM_2.5_-induced ROS and inhibit the subsequent activation of p38 mitogen-activated protein kinases by ROS - phenanthrene diminished vascular protein leakage, leukocyte infiltration, and pro-inflammatory cytokine release within the BALF
*Lonicera japonica* Wall. (Caprifoliaceae) [233]	N/A	30% ethanol	20–100 mg/kg, oral	BALB/c mice	PM_2.5_, 500 μg/m^3^ in a chamber for 5 h/day for 12 weeks	- diminishing levels of T cells (CD4+ T cells, CD8+ T cells, and total Th2 cells) - reducing IgG and IgE levels - enhancing pulmonary antioxidant defense by regulating SOD activity, GSH, and MDA content
*Lycium chinense* Mill. (Solanaceae) [234]	RT	30% ethanol	200–400 mg/kg, oral	male BALB/c mice	PM_10_, 20 mg/mL, inhalation	- suppressing the upregulation of IL-4, IL-13, and TNF-α and COX2 expression through the inhibition of MAPKs (ERK and JNK) signaling - reducing the serum levels of IgE and the expression of TNF-α and Bax in lung tissues
*Mentha × piperita* L. (Lamiaceae) [235]	N/A	essential oil	0.1 *v*/*v* %, nasal inhalation	female BALB/c mice	PM_10_, 100 μg in 50 μL saline, intranasal instillation	- diminishing IL-6 levels, along with a decrease in pro-inflammatory and T helper 2-specific cytokines - suppressing the phosphorylation of JAK2 and STAT3
*Opuntia ficus-indica* (L.) Mill. (Cactaceae) [236]	ST	8 volume of water, 30% ethanol, or 50% ethanol	200 mg/kg, oral	male BALB/c mice	PM_10_, 3 mg/mL, intranasal instillation	- reducing neutrophil infiltration and immune cell counts (CD3+/CD4+, CD3+/CD8+, and Gr-1+/CD11b) in BALF and lung tissue - diminishing the expression of cytokines and chemokines, such as CXCL-1, IL-17, MIP-2, TNF-α, COX-2, IL-1α, IL-1β, IL-5, IL-6, TRPV1, and MUC5AC - preventing the accumulation of IRAK-1, TNF-α, and CXCL-1 in BALF and lungs - reversing histopathological damage in the trachea and lungs
*Panax ginseng* Meyer (Araliaceae) black ginseng [237]	RT	80% ethanol	0.05–0.6 g/kg, oral	male Balb/c mice	PM_2.5_, 1 mg/kg, intratracheal injection	- neutralizing ROS and suppressing ROS-mediated activation of p38 mitogen-activated protein kinase (MAPK) - maintaining the activation of Akt
*Panax ginseng* Meyer (Araliaceae) red ginseng [238]	RT	distilled water	100 mg/kg, oral	male Balb/c mice	PM_2.5_, 500 μg/m^3^, for 5 h/day for 12 weeks	- preventing pulmonary fibrosis by modulating the TGF-β1 signaling pathway - mitigating pulmonary and cognitive impairment by modulating systemic inflammation and apoptosis through the NF-κB/JNK pathway
*Platycodon grandiflorus* (Jacq.) A.DC. (Campanulaceae) [239]	RT	ultrapure water	100–200 mg/kg, oral gavage	male C57BL/6 mice	PM_2.5_, 5 mg/mL, tracheal drip	- diminishing inflammatory factor levels in bronchoalveolar lavage fluid and augmented antioxidant capacity in lung tissue - lowering lung cell apoptosis - mitigated acute lung injury (ALI) by regulating the STAT3, JUN, and AKT1 signaling pathways
*Pyrus nivalis* Lindl. (Rosaceae) [240]	FT	deionized water + 400 g of 1% citric acid (Final volume = 125 mL)	60–120 μL, oral	female BALB/c mice	PMs, 100 μg in 30 μL of phosphate-buffered solution, intratracheal instillation	- enhancing phagocytic activity of phagocytes and increasing differentiation of M1-dominant macrophages in the lungs - augmenting the phagocytic function of airway phagocytes
*Pyrus pyrifolia* (Burm.f.) Nakai [241]	FT	N/A [241]	100–400 mg/kg, oral	male Balb/c mice	PM_2.5_, 1 mg/kg, intranasal instillation	- exhibiting anti-inflammatory and mucolytic properties, attributed to increase levels of lung substances P and ACh and reduce expression of lung MUC5AC and MUC5B mRNA
	FT	purified water [242]	100–400 mg/kg, oral	male Balb/c mice	PM_2.5_, 1 mg/kg, intranasal instillation	- augmenting serous fluid production in lung tissue - elevating substance P and ACh levels - inhibiting mucus-production-related expression of MUC5AC and MUC5B mRNA - inhibiting the upregulation of PI3K/Akt and p38 MAPK mRNA expression
*Rosa laevigata* Michx. (Rosaceae) [243]	FT	distilled water [244]	15.625–250 μg/mL	human lung epithelial A549 cells	PM_10_, 25–400 μg/mL	- mitigating PM_10_-associated cytotoxicity - reducing the expression levels of the MAPK/NF-κB pathways and its downstream effector, COX-2 - suppressing the expression of TNF-α, IL-1β, IL-6, IL-13, and IL-17 at the mRNA level
*Schisandra chinensis* (Turcz.) Baill. (Schisandraceae) [245]	FT	70% ethanol	200–400 μg/mL	murine macrophage RAW 264.7 cell	PM_2.5_, 50 μg/mL	- blocking the release of NO and PGE2 - suppressing the increase in IL-6 and IL-1β expression, resulting in reduced extracellular release - reducing the movement of NF-κB into the nucleus.
*Securinega suffruticosa* (Pall.) Rehder (Phyllanthaceae) [246]	LF	80% ethanol	100–300 mg/kg, oral	male C57/BL6 mice	PM_2.5_, 20 mg/kg, inhalation	- lowering the levels of C-X-C motif ligand 1/2 in BALF - reducing the expression of Muc5ac, ICAM-1, TNF-⍺, and IL-6 mRNA - inhibiting the expression of cell adhesion molecules (CAMs), TNF-⍺, and inflammasome markers, e.g., NLRP3, caspase-1, and ASC - suppressing PM_2.5_-mediated generation of intracellular ROS and preventing the translocation of NF-κB p65 into the nucleus in human umbilical vein endothelial cells
*Thalictrum minus* L. (Ranunculaceae) [247]	AP	water	10–40 mg/kg, oral	male C57 mice	PMs, 50 mg/kg, intratracheal injection	- decreasing total protein in BALF, and effectively suppressing PM-induced increases in leukocytes and macrophages in BALF - suppressing MPO activity in lung tissues - decreasing inflammatory cytokines such as TNF-α, IL-6, and IL-1β - reducing NO levels, and increasing SOD activity in BALF - promoting the expression of p-AMPK-Nrf2 and suppressing the expression of KEAP, preventing the activation of the MAPKs-NLRP3/caspase-1 and COX2 pathways and inhibiting apoptotic pathways
*Trigonella foenum-graecum* L. (Fabaceae) [248]	SD	30% ethanol	50 and 200 mg/kg, oral	female BALB/c mice	PMs, 0.1 mg/25 μL, intratracheal injection	- diminishing weight gain of the liver, lung, and spleen - reducing IgE, IL-6, and IFN-γ levels, and decreased bronchial mucus secretion and smooth muscle thickness

RT: root; RZ: rhizome; TB: tuber; SP: sprout; PL: peel; N/A: not available; ST: stem; FT: fruit; LF: leaf; AP: arial part; SD: seed.

#### 3.2.2. Herbal Medicines for Cardiovascular Systems

Chronic exposure to fine PM air pollution has been linked to a heightened risk of developing cardiovascular disease (CVD) and premature death from cardiovascular causes [263,264]. Evidence from cohort studies suggests that prolonged exposure to PM_2.5_ can initiate and accelerate conditions such as atherosclerosis, hypertension, and type 2 diabetes mellitus, all of which are associated with a greater risk of adverse cardiovascular events and a subsequent reduction in life expectancy [265,266,267,268]. Researchers are actively exploring innovative preventive and therapeutic strategies to safeguard human health, with a particular focus on the use of herbal remedies and traditional herbal formulations. In 2019, an aqueous extract of rooibos (*Aspalathus linearis* (Burm.f.) R.Dahlgren (Fabaceae)) was investigated for its cardiovascular protective effects in Wistar rats exposed to a methanol extract of diesel exhaust particles (DEPs). The study revealed that DEPs increased malondialdehyde (MDA) and conjugated diene (CD) levels in the aorta and heart, while also inducing IL-8, TNFα, IL-1β, NF-κB, and IκKB expression and reducing IL-10 and IκB mRNA levels. These effects were reversed using the rooibos extract, which also mitigated the DEP-induced elevation of Nrf2 and HO-1 mRNA levels [269]. Sriram and colleagues investigated the effectiveness of fish omega-3 polyunsaturated fatty acids (ω-3 PUFAs) in reducing PM-induced inflammation and vasoactivity in human vascular endothelial cells [270]. Their study showed that pretreatment with ω-3 PUFAs mitigated the PM-induced release of pro-inflammatory cytokines, IL-6 and IL-8, as well as the release of vasoconstrictor endothelin-1 (ET-1). Jiao et al. revealed that 2-undecanone, extracted from *Houttuynia cordata* Thunb. (Saururaceae), induces the expression of the antioxidant enzyme HO-1 by activating Nrf2, thereby suppressing the NF-κB pathway and mitigating inflammatory damage to the mouse myocardium caused by PM_2.5_ exposure [271]. *Flueggea suffruticosa* (Pall.) Baill. or *Securinega suffruticosa* (Pall.) Rehder (Phyllanthaceae) exhibited significant protective effects by suppressing the diesel exhaust particle (DEP)-induced elevation of CAMs, TNF-α, and inflammasome markers (NLRP3, caspase-1, and ASC) in the thoracic aorta. It also inhibited PM_2.5_-induced ROS production and prevented NF-κB p65 nuclear translocation in human umbilical vein endothelial cells [246]. In addition, highly effective anti-inflammatory and antioxidant herbs were investigated for their protective and therapeutic effects against symptoms caused by exposure to PM. For example, a polyphenol-enriched supplement containing extracts from *Lippia citriodora* Kunth (or *Aloysia citrodora* Paláu (Verbenaceae), *Olea europaea* L. (Oleaceae), *Rosmarinus officinalis* L. or *Salvia rosmarinus* Spenn. (Lamiaceae), and *Sophora japonica* L. or *Styphnolobium japonicum* (L.) Schott) (Fabaceae) significantly reduced pollutant-induced ROS production in human endothelial cells and positively influenced cardiovascular health. Its beneficial effects, including counteracting pollution-induced heart rate changes in medaka embryos, are likely due to its inhibition of the AhR receptor response and reduction in ROS generation [272]. Furthermore, a clinical trial conducted in Wuhan, China, investigated the efficacy of a herbal blend in mitigating cardiopulmonary damage induced by PM_2.5_ air pollution in adults [273]. A herbal blend (GLP) containing ginseng (*Panax ginseng* C.A.Mey. (Araliaceae)), lilii bulbus (from a perennial plant in the *Lilium* genus), and poria (*Poria cocos* F.A.Wolf or *Wolfiporia extensa* (Peck) Ginns (Polyporaceae)) was administered to 120 participants residing in Wuhan, China, who were exposed to PM for three months (November 2018 to January 2019). This study found that, compared to the placebo group, the GLP group exhibited significantly increased antioxidant biomarkers, including SOD and paraoxonase1 (PON1), along with elevated nitric oxide and club cell secretory protein (CC16) levels, while the expression of IL-6, an inflammatory marker, was significantly reduced. Several studies have explored the effects of traditional herbal medicine in alleviating symptoms caused by PM exposure. Researchers from the Institute of Chinese Materia Medica, China Academy of Chinese Medical Sciences, Beijing, investigated the protective effects of the Chinese herbal Shengmai formula—comprising Ginseng radix et Rhizoma Rubra (*Panax ginseng* C.A.Mey.), Ophiopogonis Radix (*Ophiopogon japonicus* (Thunb.) Ker Gawl. (Asparagaceae)), and Schisandrae Chinensis Fructus (*Schisandra chinensis* (Turcz.) Baill. (Schisandraceae)) in a 1:2:1 (*w*/*w*) ratio—against myocardial injury following exposure to ultrafine particles (PM_0.1_) in rat and H9C2 cell models [260]. This study demonstrated that the Shengmai formula effectively prevented oxidative stress and protected against myocardial injury caused by ultrafine PM and myocardial ischemia in rats, likely through the involvement of the PI3K/AKT/p38 MAPK/Nrf2 signaling pathway. The following year, researchers from the same institute studied the effects of the Shengmai formula on diesel exhaust particle-induced atherosclerosis and cardiac dysfunction in a myocardial infarction model, finding that the Shengmai formula effectively treated dyslipidemia, atherosclerosis, myocardial ischemia, and oxidative stress in ApoE^−^/^−^ mice to varying degrees [274]. Another Traditional Chinese Medicine formula, Shenlian, which contains extracts of *Salvia miltiorrhiza* Bunge (Lamiaceae) and *Andrographis paniculata* (Burm.f.) Nees (Acanthaceae) (comprising 3% tanshinone II A, 38% salvianolic acid B, and 20% andrographolide), was studied for its effects on ultrafine particle-induced myocardial ischemic injury in rats [275]. The findings revealed that Shenlian inhibited NLRP3 inflammasome activation in the NOD-like signaling pathway and reduced the release of IL-1 family cytokines. Kyung-Ok-Ko, a traditional oriental prescription composed of *Rehmannia glutinosa* var. *purpurae* (Makino) Makino and Nemoto (Orobanchaceae), *Lycium chinense* Mill. (Solanaceae), *Aquilaria agallocha* (Lour.) Roxb. ex Finl. or *A. malaccensis* Lam. (Thymelaeaceae), *Poria cocos* F.A.Wolf, *Panax ginseng* C.A.Mey., and honey, was evaluated to assess its effects on suppressing PM_2.5_-induced vascular barrier disruption [257]. The formula was found to significantly scavenge PM_2.5_-induced ROS, inhibit the ROS-induced activation of p38 MAPK, and activate Akt, thereby maintaining endothelial integrity and potentially protecting against PM-induced vascular hyperpermeability.

#### 3.2.3. Herbal Medicines for Skin

Skin, particularly the stratum corneum, functions as a crucial physical barrier that is constantly exposed to external stresses, including ROS produced from PM, such as PM_2.5_. Human dermal fibroblasts and immortalized keratinocyte (HaCaT) cells are vulnerable to inflammatory responses triggered by air pollutants through the induction of pro-inflammatory cytokines and ROS. These pollutants further compromise the skin barrier, facilitating deeper penetration into the epidermis and exacerbating harmful effects. Prolonged exposure to PM_2.5_ can lead to cumulative skin damage, contributing to premature aging, hyperpigmentation, and acne, and increasing the risk of conditions such as psoriasis, atopic dermatitis, and even skin cancer. [123,276,277]. Plants and phytochemicals with potent antioxidant and anti-inflammatory properties—such as *Artocarpus altilis* [278], *Phellodendron amurense* [276], *Camellia japonica* [279], polysaccharides from Gogi berry [280] and punicalagin from pomegranate [218]—have attracted significant interest due to their potential to prevent skin disruption and disease caused by long-term exposure to PM-induced oxidative stress and inflammation, as shown in Table 4 and Table 5. Several of these plants, including *Hamamelis virginiana* (witch-hazel) [281], Siegesbeckiae Herba [187,188] and Astragali Radix [215], have been traditionally used for skin treatment. Notably, some herbs listed in Table 4 and Table 5 exhibited outstanding protective effects in comparative studies. For instance, the Siegesbeckiae Herba extract (SHE) was found to be the most effective at defending against PM_10_ cytotoxicity among 23 medicinal plant extracts examined [187], while *Ecklonia cava* showed superior protective effects among 50 marine algae [282].

HaCaT keratinocytes are frequently used in in vitro models to investigate the impact of PM on the skin and to evaluate the biological effects of plant extracts, particularly their antioxidant and anti-inflammatory activities, in preventing PM-induced skin damage. Several studies have identified the active constituents responsible for protective effects against PM. Phenolic compounds, in particular, have been highlighted for their key role in skin protection due to their strong antioxidant activity [283]. Examples include resveratrol from grapes [284], (−)-epigallocatechin gal-late (EGCG) from green tea [285,286], chlorogenic acid from Siegesbeckiae Herba extract [187], 4-O-feruloylquinic acid from Phellodendron amurense [276], and Formononetin (FMT), an isoflavone found in Astragali Radix [287]. These compounds play a crucial role in defending the skin against oxidative stress and inflammation caused by PM exposure.

Interestingly, some studies have reported that isolated phytochemicals demonstrate superior activity compared to crude extracts. For instance, Formononetin significantly upregulated the expression of KRT 17, a marker related to keratinocyte proliferation that is reduced upon PM exposure, while the crude Astragali Radix extract had no effect on KRT 17 expression [287]. However, in some cases, active compounds are present in small amounts, making it a challenge to achieve effective doses. As a result, the skin-protective effects often rely on the synergistic interactions of various constituents within the herbs, as demonstrated in study by Jiyoung Choi et al. (2021) on the protective effect of *Hamamelis virginiana* and its active compound, hexagalloylglucose (HGG). Previous studies on the skin-protective effects of natural products, exercised through various pathways, have suggested that these compounds can serve as valuable agents in the dietary supplement or cosmetic industries, combating PM-induced skin damage. However, ex vivo and in vivo studies on plant extracts and phytochemicals remain limited. Further research is necessary to evaluate the efficacy and toxicity profiles of these promising natural products in animal models in order to better understand their potential for skin protection.

**Table 4:** Plant extracts targeting PM-induced skin damage in in vitro and in vivo studies.

Sources	Part Used	Solvent	Type of Studies	Model	PM Types and Administration	Biological Results
*Artocarpus altilis* [278]	heartwood	methanol	in vitro	HaCaT keratinocyte	Urban dust PM (50 μg/cm^2^)	- antioxidant effects (reducing reactive oxygen species (ROS) and the lipid peroxidation marker, 4-hydroxynonenal - anti-inflammatory effects (downregulating pro-inflammatory mediators, including TNF-α, TNFR1, and COX-2 and modulating the MAPK signaling pathway) - restoring skin barrier integrity by enhancing the expression of essential barrier proteins, including filaggrin, loricrin, and aquaporin 3
Siegesbeckiae Herba (*Sigesbeckiae orientalis* L, *Sigesbeckiae pubescens* Makino or *Sigesbeckiae* glabrescens Makino) [187]	leaf	Hot water at 90 °C	in vitro	HaCaT keratinocytes	Standardized fine dust (PM_10_-like)	- mitigating the PM_10_-induced death of HaCaT cells - reducing LDH release - antioxidant effects (reducing ROS and lipid peroxidation and promoting the synthesis and recycling of glutathione (GSH), activating the NRF2 system in cells by upregulating protective genes, such as HMOX1 and NQO1, and decreasing the expression of KEAP1, the negative regulator of NRF2)
Siegesbeckiae Herba (*Sigesbeckiae orientalis* L, *Sigesbeckiae pubescens* Makino or *Sigesbeckiae* glabrescens Makino) [188]	leaf	Hot water at 90 °C	in vitro	human sebocytes and outer root sheath (ORS) cells	PM_10_	- antioxidant effects (reducing ROS level by inhibiting aryl hydrocarbon receptor (AhR)) - anti-inflammatory effects (suppressing inflammatory cytokine and NF-kB expression, and inhibiting MMP activity) - reducing the production of sebum
*Phellodendron amurense* [276]	bark	50% ethanol	in vitro	HaCaT cells	Diesel particle matter (DPM), as a model of PM_2.5_	- inhibiting DPM-induced Ca^2+^ influx through the proteinase-activated receptor-2 (PAR-2) signaling pathway - restoring the DPM-induced skin barrier damage by upregulating tight-junction proteins such as Zonula Occludens-1 (ZO-1) and Occludin
*Hamamelis virginiana* [281]	stem and leaf	40% 1,3-butylene glycol	in vitro	HaCaT cells	DPM, as a model of PM_2.5_	- mitigating PM-induced intra- cellular calcium influx - reducing the activation of inflammatory markers such as NF-κB - restoring skin barrier function by upregulating tight-junction proteins such as Occludin
*Ecklonia cava* Kjellma [288]	alga	80% (*v*/*v*) aqueous ethanol	in vitro	HaCaT cells	PM_10_-like fine dust (3–100 μg mL^−1^)	- antioxidant effect (reducing lipid peroxidation) - improving cell viability - anti-inflammatory effects (downregulating the expression of microsomal PGE2, COX-1 and COX-2) - antioxidant (lowering ROS levels) - improving cell viability
[282]	alga	80% (*v*/*v*) aqueous ethanol		HaCaT cells	PM_10_-like fine dust (25–400 μg mL^−1^)	
*Astragalus mongholicus* Bunge (Astragali Radix) [287]	root	ethanol	in vitro	- HaCaT cells - 3D skin reconstructed model	diesel particulate matter (DPM) (200 μg/mL)	- promoting keratinocyte proliferation by increasing Ki67 and keratin (KRT) 16 expression - reducing involucrin levels, the keratinocyte differentiation markers - inhibiting apoptosis by downregulating the expression of apoptotic protein; cleaved caspase-3, p53, and Bax - inhibiting apoptosis and improving skin proliferation through ERK signaling suppression, part of the MAPK pathway
*Camellia japonica* [279]	flower	70% (*v*/*v*) ethanol	in vitro	normal human dermal fibroblasts (NHDFs)	Urban dust	- strong antioxidant properties (inhibited the production of ROS, MMP-1 expression, and a xenobiotic response element (XRE)-luciferase activity by suppressing AhR signaling pathway) - decreasing production of malondialdehyde (MDA), a lipid peroxidation marker - improving skin structure and promoting collagen synthesis
			ex vivo	skin explants

**Table 5:** Phytochemicals targeting PM-induced skin damage in in vitro and in vivo studies.

Phytochemicals	Sources	Type of Studies	Model	PM Types and Administration	Biological Results
Eupafolin (flavonoid) [289]	*Phyla nodiflora*	in vitro	human keratinocyte HaCaT cells (pretreatment with 10 µM eupafolin)	PM (Standard Reference Material^®^ 1649b) 50 g/cm^2^	- inhibiting PM-induced COX-2 protein, gene expression, and PGE2 production - antioxidant effects (inhibiting NADPH oxidase activity and ROS production) - anti-inflammatory effects (inhibiting PM-induced MAPK phosphorylation and downregulating NF-κB signaling pathways) - anti-inflammatory effects (inhibiting COX-2 expression and decreasing infiltrating inflammatory cells) - inhibiting PM-induced hyperproliferation of epidermis
		in vivo	BALB/c nude mice (pretreatment with 10 µM eupafolin)	topical treatment with PM	
Resveratrol, Resveratryl Triacetate (polyphenol) [284]	Grapes, berries, peanuts, etc.	in vitro	human epidermal keratinocytes (HEKs)	PM_10_	- antioxidant effect (reducing ROS production) - anti-inflammatory effects (inhibiting pro-inflammatory cytokines, such as IL-6 and TNF-α)
Punicalagin [285] (–)-Epigallocatechin-3-Gallate [285]	Pomegranate Green tea	in vitro	human epidermal keratinocytes	PM_10_	- potent antioxidant effect (reducing PM-induced ROS level and expression of NADPH oxidases (NOX-1, NOX-2) - anti-inflammatory effects (downregulating the expression of TNF-α, IL-1β, IL-6, IL-8, and MMP-1)
(−)-epigallocatechin gallate (EGCG) [286]	Green tea	in vitro	human dermal fibroblasts (HDFs)	Fine dust particles (FDP) (ERM-CZ100, PM_10_-like) (10 mg/mL)	- antioxidant effect (reducing intracellular ROS) - improving cell viability in HDFs exposed to fine dust particles - inhibiting elastase and collagenase activity which may be useful in preventing FDP-induced skin aging - suppressing the expression of matrix metalloproteinases (MMPs) via NF-κB, AP-1, and MAPK signaling pathways
Polysaccharides [280]	Gogi berry *Lycium barbarum*	in vitro	HaCaT cells	PM_2.5_ (100 μg/mL)	- modulating several stress-related pathways (lowering intracellular ROS levels and enhancing the activity of antioxidant enzymes) - reducing endoplasmic reticulum (ER) stress (downregulating stress markers like GRP78 and CHOP) - inhibiting PM_2.5_-induced autophagy and mitochondrial damage
chlorogenic acid [187]	Siegesbeckiae Herba extract	in vitro	HaCaT keratinocytes	Standardized fine dust (PM_10_-like)	- improved the survival rate of HaCaT cells exposed to PM_10_ (by activating NRF2 system) - inhibiting PM-induced LDH release - antioxidant effects (reducing ROS production and promoting the synthesis and recycling of glutathione (GSH))
4-O-feruloylquinic acid (FQA) [276]	*Phellodendron amurense*	in vitro	HaCaT cells	DPM, as a model of PM_2.5_	- inhibiting Ca^2+^ influx induced by DPM by regulating PAR-2 signaling pathway
hexagalloylglucose (HGG) [281]	*Hamamelis virginiana*	in vitro	HaCaT cells	DPM, as a model of PM_2.5_	- reducing Ca^2+^ influx induced by DPM
Eckol Dieckol [288]	*Ecklonia cava*	in vitro	HaCaT cells, human epidermal keratinocytes	PM_10_-like fine dust (3–100 μg/mL)	- improving cell viability - antioxidant activity (reducing lipid peroxidation) - anti-inflammatory activity (suppressing the expression of inflammatory cytokines, including TNF-α, IL-1β, IL-6, and IL-8) - antioxidant effect (reducing ROS generation and lipid peroxidation) - improving cell viability and decreasing apoptotic cell death - preventing PM_2.5_-Induced Mitochondrial damage - anti-inflammatory activity - (inhibiting the activation of the MAPK signaling pathway) - reducing apoptosis-related protein levels by decreasing proapoptotic protein-Bax, antiapoptotic protein-Bcl-2, and cleaved caspase-3 - anti-inflammatory activity - (decreasing PM_10_-induced PGE2 production and suppressing the expression of COX-1, COX-2, and mPGES-1) - improving cell viability - reducing morphological change induced by PM in 3D-skin model
Eckol [290]	*Ecklonia cava*	in vitro	human HaCaT keratinocytes	PM_2.5_ (25 mg/mL)	
Dieckol [282]	*Ecklonia cava*	in vitro	human keratinocytes (HaCaT cells)	PM_10_-like fine dust (25 to 400 μg/mL)	
Afzelin [291]	*Thesium chinense* Turcz	in vitro	HaCat cells	PM (25 µg/cm^2^)	- antioxidant effect (reducing PM-induced oxidative stress by inhibiting the generation of ROS and suppressing the activation of the p38 MAPK and AP-1 pathways) - anti-inflammatory effect (downregulating pro-inflammatory cytokines including IL-1α, IL-1β and IL-6 induced by PM)
Formononetin [287]	*Astragalus mongholicus* Bunge (Astragali Radix)	in vitro	- HaCaT cells 3D skin reconstructed model	diesel particulate matter (DPM) (200 μg/mL)	- promoting keratinocyte proliferation by increasing Ki67, KRT16 and KRT17 expression - reducing involucrin levels, the keratinocyte differentiation markers - inhibiting apoptosis by downregulating the expression of apoptotic protein; cleaved caspase-3, p53, and Bax
Diphlorethohydroxycarmalol (DPHC)	*Ishige okamurae*	in vitro	Human keratinocytes (HaCaT cells)	PM_2.5_	- antioxidant effects (reducing ROS production and mitochondrial ROS) - decreasing mitochondrial Ca^2+^ level and balancing membrane permeability - suppressing endoplasmic reticulum (ER) stress, and autophagy - inhibiting the activation of MAPK signaling pathway and reducing PM_2.5_ -induced apoptosis - protecting DNA and skin damage from oxidative stress by inhibiting lipid peroxidation and protein carbonylation) - reducing epidermal height induced by PM_2.5_
		in vivo	HR-1 hairless mice	PM_2.5_	

Based on extensive scientific research on various herbs and their active compounds to mitigate PM-induced skin impairment, researchers have increasingly focused on developing bioactive agents or formulations for application in dietary supplements and skincare products. Eupafolin, a flavonoid derived from Phyla nodiflora, has been recognized for its antioxidant, anti-inflammatory, and skin-protective effects against PM_2.5_ exposure, as previously mentioned in Table 5 [289,292]. In 2016, Zih-Chan Lin et al. synthesized Eupafolin nanoparticles (ENDSs) and evaluated their solubility, skin absorption, and protective effects on HaCaT keratinocytes exposed to PM. ENDSs exhibited superior activity compared to raw eupafolin, significantly reducing oxidative stress by lowering ROS levels and NADPH oxidase activity. They also suppressed inflammation by downregulating COX-2 expression and PGE2 production through the inhibition of the MAPK and NF-κB signaling pathways. Furthermore, ENDSs enhanced solubility and skin penetration, underscoring their potential as a promising therapeutic agent for preventing PM-induced skin damage [293]. Similarly, Pao-Hsien Huang et al. (2018) developed nanoparticles of 7,3′,4′-trihydroxyisoflavone (734THI), an active agent derived from daidzein in soybeans, which has demonstrated various skin benefits. The 734THI nanoparticles exhibited enhanced solubility and cellular uptake, significantly reducing inflammation by inhibiting COX-2 and MMP-9 expression, while downregulating the MAPK signaling pathway in HaCaT keratinocytes exposed to PM [294].

Several functional foods with antioxidant properties have also been studied for their protective effects against PM-induced skin conditions. Seong Min Hong et al. (2021) evaluated the effect of fermented blueberry and black rice extract (FBBBR), containing Lactobacillus plantarum MG4221, on skin inflammation and barrier dysfunction triggered by PM_2.5_. In HaCaT keratinocytes, FBBBR significantly reduced levels of pro-inflammatory cytokines (IL-1β, IL-6, IL-8) by inhibiting the NF-κB and MAPK signaling pathways, which are commonly activated by air pollutants. The anti-inflammatory effects of the extract were attributed to its active components, syringic acid and kuromanin, which effectively decreased cytokine levels. In an in vivo model, the oral administration of FBBBR alleviated symptoms of atopic dermatitis (AD), such as transepidermal water loss, erythema, and scratching behaviors. The treatment also enhanced the expression of skin barrier proteins, including filaggrin and involucrin, and reduced serum IgE levels and T helper 2-associated cytokines, indicating its potential as a functional food for managing pollution-induced skin disorders [278].

#### 3.2.4. Herbal Medicines for Neurological System

PM can enter the brain via the bloodstream, causing damage to hippocampal tissue and disrupting neuronal synapse function from the olfactory bulb to the brain. These effects can lead to memory and learning impairments and, eventually, neurological disorders, as mentioned earlier. Ju Hui Kim et al. (2023) reported that Korean red ginseng (Panax ginseng) exerted neuroprotective effects by regulating the antioxidant system and restoring mitochondrial function compromised by PM_2.5_ toxicity. Additionally, the Korean red ginseng extract alleviated PM_2.5_-induced cognitive impairment by modulating systemic inflammation and apoptosis through the NF-κB/JNK signaling pathway [238]. This neuroprotective effect can be attributed to ginsenosides, bioactive compounds found in Panax ginseng, which inhibit oxidative stress and apoptosis while enhancing cognitive function [295].

#### 3.2.5. Herbal Medicines for Gastrointestinal (GI) System

Recent research has highlighted the impact of PM_2.5_ on the GI system, demonstrating that it can penetrate the GI tract via mucociliary clearance and the oral route [296]. High concentrations of PM induce oxidative stress, leading to GI epithelial cell death, inflammation, the disruption of tight-junction proteins, increased gut permeability, impaired intestinal immune barrier function, and the formation of toxic byproducts that exacerbate intestinal injury [296,297]. This exposure disrupts the gut microbiome, potentially leading to inflammation and gastrointestinal conditions such as inflammatory bowel disease (IBD), ulcerative colitis (UC), and Crohn’s disease (CD) [161,298]. A Chinese herbal extract blend, Fresh Clear (FC)—consisting of *Lonicera japonica*, *Momordica grosvenori*, and broccoli seed extracts with chlorogenic acid, mogroside V, and glucoraphanin as their respective active compounds—has been found to downregulate the expression of pro-inflammatory cytokines (IL-1β, IL-6, IL-8, and TNF-α) in A549 and THP-1 cells exposed to PM_2.5_, but upregulate tight-junction protein mRNA expression in A549 cells. Additionally, in an in vivo model, FC demonstrated anti-inflammatory properties by upregulating the levels of IL-10 and the tight-junction protein ZO-1 while downregulating COX-2 expression in response to PM_2.5_ exposure [297]. These pharmacological effects might be associated with the anti-inflammatory properties of the individual herbs and active ingredients in the formula. For instance, Lonicera japonica and Mogroside V, found in *Momordica grosvenori*, have been shown to reduce the expression of inflammatory factors (TNF-α, IL-1β, IL-6 and IFN-γ) through multiple signaling pathways. Additionally, glucoraphanin present in broccoli seed extracts can be metabolized into sulforaphane, which activates the Nrf2 signaling pathway [297]. This evidence suggests that combining multiple herbs in the polyherbal formulation could enhance its therapeutic potential, offering synergistic protection and facilitating recovery from PM_2.5_-induced damage.

#### 3.2.6. Herbal Medicines for Ocular Health

Previous studies have demonstrated that exposure to PM can damage the corneal and conjunctival epithelium through mechanisms such as oxidative stress, inflammation, and autophagy. These processes contribute to inflammatory responses, dry eye-like symptoms, and the development of ocular corneal diseases [164,165,167]. Moreover, PM exposure can impair cell migration and delay the corneal epithelium wound healing process [168]. *Peucedanum japonicum* Thunberg extract (PJE) has been shown to promote corneal wound healing and re-epithelialization while maintaining corneal thickness following ultrafine particulate matter (UPM)-induced damage to the ocular surface in in vitro and in vivo studies. The activity of PJE is believed to be linked to its antioxidant and anti-inflammatory properties. In these studies, PJE demonstrated free radical scavenging activity and significantly upregulated the expression of antioxidative genes, including CAT, HO1, and GPX1, although SOD1 expression remained unchanged. The major components detected in PJE—chlorogenic acid (CA), neochlorogenic acid (NCA) and cryptochlorogenic acid (CCA)—were individually effective in improving the wound healing and cell migration and synergistically enhanced human corneal epithelial cell (HCEC) migration. It was suggested that PJE, a natural product, may be a potential candidate for the treatment of corneal injuries associated with inflammation and oxidative stress [299]. For dry eye disease (DED), Achyranthis radix hot water extract (ARE) has shown potential in alleviating symptoms caused by exposure to UPM in a rat model. ARE promoted tear secretion and improved corneal surface irregularities. Furthermore, ARE protected against corneal epithelial cell death caused by UPM exposure, increased rMuc4 expression in the cornea, and enhanced goblet cell density in the conjunctiva. These findings highlight its potential as a therapeutic agent for UPM-induced DED. Although the bioactive compound responsible for these effects has not yet been identified, the known anti-inflammatory properties of ARE’s components, such as saponins and phytoecdysones (including ecdysterone and inokosterone), are thought to contribute to its protective role against DED inflammation [300].

## 4. Future Directions and Research Gaps

This review on herbal medicines targeting PM-induced health issues highlights their promising potential as alternative preventive and therapeutic agents. Numerous studies have highlighted the antioxidant and anti-inflammatory properties of herbal extracts and phytochemicals, which are useful in mitigating PM-induced cellular damage across various organ systems. However, most current research has primarily focused on in vitro and animal models, which may not fully reflect the complexity of human physiology or the long-term health impacts of PM exposure. To address this limitation, future research should prioritize human clinical trials to assess the safety, optimal dosages, and the efficacy of herbal treatments under long-term PM exposure. Although limited, existing human studies have provided encouraging evidence for practical applications. For instance, Chen (2024) demonstrated that Qiju granules, a Traditional Chinese Medicine formulation containing Lycium, Chrysanthemum, and Rehmannia, effectively preserved lung function against PM_2.5_-induced damage when compared to placebo group in a 4-week randomized controlled trial [204]. Similarly, Qianjinweijing decoction (QJWJ), a decoction of several Chinese herbs with potent antioxidant and anti-inflammatory properties, showed protective effects against PM_2.5_-induced lung dysfunction in a 4-week randomized, double-blinded, placebo-controlled trial [203]. Another herbal formula (GLP), containing ginseng, lilii bulbus, and poria, demonstrated anti-inflammatory and antioxidant effects, protecting cardiopulmonary health against PM_2.5_-induced damage in a randomized, double-blinded, and placebo-controlled trial [273]. Furthermore, clinical trials have confirmed the benefits of antioxidant-rich foods in counteracting PM-induced damage. For example, Nobile (2021) demonstrated the efficacy of a polyphenol-enriched dietary supplement (Zeropollution^®^) in mitigating pollution-induced skin damage after 2 weeks’ intake [301]. Similarly, dietary antioxidant intake is associated with cardiovascular protection following PM_2.5_ exposure, as indicated by improved pulse and blood pressure metrics [302].

To advance the promising potential of herbal medicines highlighted in this review, it is worth exploring whether these herbs can provide protective effects across multiple organ systems, potentially offering broad-spectrum protection. Herbal medicines may vary in effectiveness depending on the target organ system. Since long-term consumption may be required for sustained protection against PM-induced damage, concerns regarding the potential side effects or toxicity of herbal compounds at higher doses must be addressed. Extensive research is required to assess the long-term safety and toxicity profiles of these herbs while examining the active compounds found in specific plants. For instance, a 12-month study on male and female Sprague Dawley rats consuming Korean Red Ginseng demonstrated its safety [303]. Similarly, research on the long-term oral administration of ursolic acid revealed no toxic effects [304]. Conversely, the long-term intake of resveratrol was reported to function as a thyroid disruptor and a goitrogen [305], while the prolonged oral administration of Siegesbeckiae Herba in rats resulted in toxic effects, including reduced body weight gain, liver damage, and lung injury [306]. However, the current publication of research remains limited. Further studies are essential to elucidate the precise molecular mechanisms, such as the roles of these compounds in regulating gene expression and signaling pathways, particularly in mitigating PM-induced damage. This understanding could reveal targeted therapeutic approaches.

Additionally, future research should focus on PM’s impact on vulnerable groups, such as children, the elderly, and those with preexisting health conditions, and explore synergistic formulations of herbal compounds to enhance bioavailability and therapeutic efficacy against PM-induced damage. Such research would contribute to a broader understanding of their applications and provide valuable insights into public health. Finally, the development of effective herbal-based products, whether as supplements, medicines, or cosmetics containing optimal doses of active ingredients, could offer a promising alternative for future healthcare.

## 5. Conclusions

Fine (PM_2.5_) and ultrafine (PM_0.1_) particulate matter pose significant health risks due to their capacity to penetrate deep into the body, leading to a range of pathological effects. Prolonged PM exposure adversely impacts various systems—respiratory, cardiovascular, skin, neurological, gastrointestinal, and ocular—emphasizing the critical need to mitigate exposure to these particles. Research has shown that the harmful impact of PM is largely due to its toxic chemical composition and the generation of reactive oxygen species (ROS), which activate cellular pathways that lead to chronic inflammation and other health issues.

**Figure 3:**
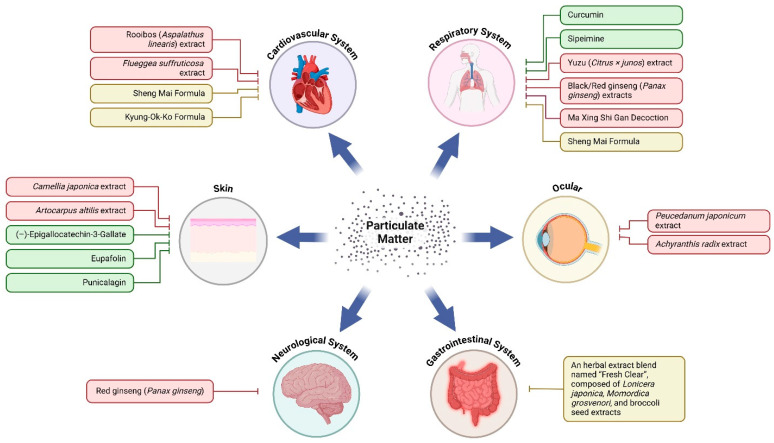
A schematic illustration summarizing the health impacts of PM exposure on human body systems and some potential natural products against PM toxicities. Rounded rectangles surrounding each body system represent natural products possessing beneficial biological properties to counteract the harmful effects of PM exposure, which green boxes indicate phytochemicals, pink boxes indicate plant extracts, and yellow boxes indicate herbal formulations. Created using BioRender (Prasansuklab, A., 2025). Available at https://BioRender.com/j55x263 (accessed on 20 November 2024).

This review underscores the potential of herbal medicines to alleviate PM-induced health issues across multiple body systems. Increasing research on herbal extracts indicates their protective effects against PM-induced cellular damage, with key mechanisms involving the inhibition of ROS production and the suppression of pro-inflammatory cytokine release. Many plant extracts and phytochemicals studied for PM-related health applications have shown efficacy in reducing oxidative stress and inflammation (Figure 3). These herbs demonstrate antioxidant properties by scavenging free radicals and enhancing cellular defense mechanisms through pathways like Nrf2, which boosts the production of antioxidant enzymes such as superoxide dismutase (SOD) and catalase. These enzymes neutralize ROS generated from PM exposure, thus protecting cellular structures from oxidative damage. Anti-inflammatory mechanisms are also essential, as numerous herbal extracts inhibit pro-inflammatory cytokines (e.g., TNF-α, IL-6) and modulate transcription factors like NF-κB, as well as signaling pathways such as NF-κB and MAPK, thereby reducing chronic inflammation, tissue damage, and apoptosis.

Air pollution and the challenge of particulate matter (PM) remain persistent global concerns that will require considerable time to address in many countries. Moreover, both short- and long-term exposure to PM are strongly associated with increased morbidity and mortality [307,308], highlighting the urgent need for effective preventive products or treatments for at-risk populations. The application of natural products, either alone or in combination with modern therapeutic approaches, could be a promising complementary strategy to mitigate the health burden of PM exposure. This review offers valuable insights into the potential herbal medicines that can be further investigated as lead compounds for drug development or applied as active ingredients in healthcare products. However, information regarding optimal dosages and their effects on humans remains limited. Continued research into the precise mechanisms, efficacy, and toxicity of herbal interventions across different systems, along with clinical studies, is essential for developing effective preventive and therapeutic solutions for affected populations.

## Figures and Tables

**Table 3:** Herbal formulations targeting PM-induced respiratory symptoms conducted in vitro and in vivo studies.

Herbal Formula	Dosage Form	Dose and Administration	Model	PM Types and Administration	Biological Results
Banhahubak [249]	tablet	200 μL of 6.29, 62.9 and 629 mg/kg, oral	female BALB/c mice	PM_10_, 100 μg, intranasal instillation	- reducing total serum IgE and IgG levels - suppressing the activation of JAK1 and STAT6 through phosphorylation - suppressing the release of pro-inflammatory cytokines and Th2-related cytokines
Biyeom-Go [250]	ointment	100% BYG, nasal inhalation	male C57BL/6 mice	PM_2.5_, 100 μg/mL, intranasal instillation	- reducing the number of goblet cells - suppressing the production of immune biomarkers (IL-33, TSLP, tumor necrosis factor alpha, and IL-8)
Bufei Huoxue [251]	capsule	0.82 g/kg, intranasal instillation	female ICR mice	PM_2.5_, 40 μg/mL, intranasal instillation	- diminishing pathological responses and inflammatory mediators, e.g., IL-4, IL-6, IL-10, IL-8, TNF-α, and IL-1β - lowering the levels of KGF, sIgA, and collagen fibers in lung tissue
Chyawanprash [252]	paste	500 and 2000 mg/kg, oral	male Balb/c mice	PM_2.5_, 100 μg/per mouse, intratracheal instillation	- suppressing the expression of inflammatory cytokines (BALF: TNF-α, IFN-γ, IL-7, IL-6 and lung: TNF-α, histamine, and IL-6), chemokines (lung: MMP-9) - reducing inflammatory cell infiltration (cell counts in BALF) and histopathological changes
Deng-Shi-Qing-Mai-Tang [253]	decoction	0.72–2.90 g/mL, oral	male and female Sprague Dawley rat	PM_2.5_, 0.5 mg/mL, intranasal instillation	- ameliorated lung tissue damage and inflammatory responses - modulating the expression of TLR4, MyD88, IKK, IκB-α, and NF-κB p65 in the TLR4/NF-κB signaling pathway - restricting the translocation of activated NF-κB into the nucleus and subsequently limits its binding to target DNA - lowering the levels of IL-1β, IL-6, IL-10, TNF-α, NO, and PGE2
Gamgil-tang [254]	decoction	100–400 mg/kg, oral	male C57BL/6 mice	PM_10_, 3 mg/mL and diesel PM, 0.6 mg/mL, intratracheal instillation	- inhibiting lung injury via the suppression of neutrophil and inflammatory mediator levels, T cell inhibition, and the amelioration of lung tissue damage - lowering neutrophilic inflammation by reducing neutrophil counts and modulating the production of neutrophil-related cytokines and chemokines (TNF-α, IL-17, MIP2, and CXCL-1)
Gwaruhaengryeon-hwan [255]	extract	100–400 mg/kg, oral	Balb/c mice	PM_10_, 3 mg/mL and diesel PM, 0.6 mg/mL, intratracheal instillation	- inhibiting inflammatory symptoms in the lungs, such as increased alveolar wall thickness, collagen fiber accumulation, and cytokine secretion - diminishing inflammatory cell infiltration in bronchoalveolar lavage fluid and lung tissue
Guo-Min [256]	decoction	10 mL/kg, gavage	Wistar rats	PM_2.5_, 10 mL/kg, intratracheal instillation	- diminishing the infiltration of neutrophils, monocytes, lymphocytes, and eosinophils - lowering the levels of pro-inflammatory mediators, MCP-1 and NE, and type 2 inflammation-related cytokines, IgE and IL-4 - suppressing goblet cell hyperplasia and the expression of MUC5AC
Kyung-Ok-Ko [257]	decoction	0.05–0.6 g/kg, oral	male Balb/c mice	PM_2.5_, 1 mg/kg, intratracheal instillation	- neutralizing ROS and suppressed ROS-mediated activation of MAPK - activating Akt, thereby preserving endothelial integrity - diminishing vascular permeability, leukocyte migration, and pro-inflammatory cytokine secretion in BALF
Ma Xing Shi Gan [258,259]	decoction	4.1–16.4 g/kg,	male Sprague Dawley rats	PM_2.5_, 0.1 mL/100 g, tracheal injection [258]	- mitigating lung injury by reducing pathological scores, lung edema, MPO activity, MDA content, levels of inflammatory factors, and CD68-positive macrophage numbers and disrupting the alveolar capillary barrier - inhibiting HMGB1/TLR4/NFκB signal pathways
				PM_2.5_, 9.2 mg/kg, intratracheal instillation [259]	- ameliorating weight loss, pathological changes, and epithelial barrier dysfunction - counteracting the TGF-β/Smad3 pathway, upregulated ZO-1 and claudin-5, and reversed the epithelial–mesenchymal transition (EMT) process
Shengma [260]	solution	1.08–4.32 mg/kg, oral	male Sprague Dawley rat	PM_0.1_, 2.0 mg in 0.3 mL of saline, tracheal instillation	- ameliorating histopathological changes induced by UFPM and significantly modified the PI3K/AKT/MAPK and oxidative stress signaling pathways - reversing the expression profiles of Cat, Gstk1, and Cyba
YiQiFuMai [261]	lyophilized injection	0.33–1.34 g/kg, tail vein injection	male C57 mice	PMs, 50 mg/kg, intratracheal instillation	- suppressing lung pathological changes and lung wet-to-dry weight ratios - diminishing MPO activity in lung tissues - lowering inflammatory cytokines, e.g., IL-1β and TNF-α - reducing NO and total protein levels in BALF - inhibiting the induction of lymphocytes in BALF - enhancing mammalian target of rapamycin (mTOR) phosphorylation and suppressing the expression of TLR4, MyD88, autophagy-related protein LC3II, and Beclin 1, and autophagy
Zangsiwei [262]	extract	1–4 mL, intragastric	male C57BL/6 mice	PM_2.5_, 2.5 mg/kg, intratracheal instillation	- downregulating the expression of TGF-βR1, Collagen II, Smad2/3, and α-SMA - suppressing the fibrotic signaling pathways - inhibiting the TGF-β/SMAD pathway, leading to decreased expression of TGF-βR1, SMAD2/3, α-SMA, and collagen type II

## Data Availability

Not applicable.
